# Identification of the remains of King Richard III

**DOI:** 10.1038/ncomms6631

**Published:** 2014-12-02

**Authors:** Turi E. King, Gloria Gonzalez Fortes, Patricia Balaresque, Mark G. Thomas, David Balding, Pierpaolo Maisano Delser, Rita Neumann, Walther Parson, Michael Knapp, Susan Walsh, Laure Tonasso, John Holt, Manfred Kayser, Jo Appleby, Peter Forster, David Ekserdjian, Michael Hofreiter, Kevin Schürer

**Affiliations:** 1 Department of Genetics, University of Leicester, Leicester LE1 7RH, UK; 2 School of Archaeology and Ancient History, University of Leicester, Leicester LE1 7RH, UK; 3 Department of Biology, University of York, York YO10 5DD, UK; 4 Institute of Biochemistry and Biology, Universität Potsdam, Karl-Liebknechtstr. 24-25, 14476 Potsdam, Germany; 5 UMR5288-CNRS/Université Paul Sabatier-Toulouse 3 Laboratoire Anthropologie Moléculaire et Imagerie de Synthèse Faculté de Médecine Purpam 37, allées Jules Guesde, 31073 Toulouse, France; 6 Research Department of Genetics, Evolution and Environment, University College London, London WC1E 6BT, UK; 7 Institute of Legal Medicine, Innsbruck Medical University, Muellerstraße 44, A-6020 Innsbruck, Austria; 8 Pennsylvania State University, Eberly College of Science, Thomas Bldg, #517, University Park, Pennsylvania 16802, USA; 9 School of Biological Sciences, Bangor University, Bangor LL57 2UW, UK; 10 Molecular Anthropology Laboratory, Department of Anthropology, Yale University, Yale, New Haven, Connecticut 06511, USA; 11 Department of Forensic Molecular Biology, Erasmus MC University Medical Centre Rotterdam, 3000 CA Rotterdam, The Netherlands; 12 Space Research Centre, University of Leicester, Leicester LE1 7RH, UK; 13 McDonald Institute for Archaeological Research, University of Cambridge, Cambridge CB2 3ER, UK; 14 Murray Edwards College, University of Cambridge, Cambridge CB3 0DF, UK; 15 Department of the History of Art and Film, University of Leicester, Leicester LE1 7RH, UK; 16 Centre for English Local History, University of Leicester, Leicester LE1 7RH, UK

## Abstract

In 2012, a skeleton was excavated at the presumed site of the Grey Friars friary in Leicester, the last-known resting place of King Richard III. Archaeological, osteological and radiocarbon dating data were consistent with these being his remains. Here we report DNA analyses of both the skeletal remains and living relatives of Richard III. We find a perfect mitochondrial DNA match between the sequence obtained from the remains and one living relative, and a single-base substitution when compared with a second relative. Y-chromosome haplotypes from male-line relatives and the remains do not match, which could be attributed to a false-paternity event occurring in any of the intervening generations. DNA-predicted hair and eye colour are consistent with Richard’s appearance in an early portrait. We calculate likelihood ratios for the non-genetic and genetic data separately, and combined, and conclude that the evidence for the remains being those of Richard III is overwhelming.

## Abstract (web summary)

King Richard III was a controversial English King whose remains are presumably deposited in Grey Friars in Leicester. Here the authors sequence the mitochondrial genome and Y-chromosome DNA of the skeletal remains and living relatives of Richard III and confirm that the remains belong to King Richard III.

## Graphical abstract

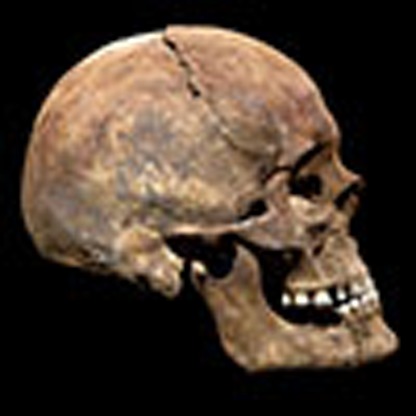

Richard III is one of the most famous and controversial English kings. His ascension to the throne in 1483, following the death of his brother, Edward IV, has been seen as contentious, involving, as it did, discrediting the legitimacy of Edward’s marriage and therefore the claim of both of Edward’s sons to the throne. Later, as yet unproven accusations arose that Richard had his two nephews murdered to solidify his own claim. Richard’s death two years later on August 22nd 1485 at the Battle of Bosworth marked the end of the Plantagenet dynasty, which had ruled for over 300 years, and the beginning of the Tudor period. Richard III was the last English king to be killed in battle, he became one of Shakespeare’s most notorious villains, and is one of the few English monarchs whose precise resting place was lost: the mystery surrounding the fate of his remains persisting to the present day.

Historical records report that after Richard III was killed on the battlefield, age 32, his remains were brought back to Leicester and buried in the medieval church of the Grey Friars^1^. The friary was dissolved in 1538 under the orders of King Henry VIII, with most of the buildings being torn down in the following years. Approximately 125 years later, a rumour arose that Richard III’s remains had been disinterred during the dissolution of the monasteries and thrown into the river Soar in Leicester^2^. However, it had long been thought that this rumour was unsubstantiated and it was therefore expected that the grave of Richard III should still lie within any remains of the Grey Friars church^3^,^4^,^5^. While historical records and the subsequent analysis thereof have long indicated the approximate location of the Grey Friars friary, and its likely situation in relation to the modern urban landscape of Leicester, the exact site of Richard III’s grave had been lost in the 527 years since his death^3^,^4^,^5^.

Although Richard III reigned for only a little over two years, substantial historical information about various features of his life and death exists. These include aspects of his physical appearance such as having a slim build, one shoulder higher than the other and that he suffered battle injuries, which resulted in his death^6^ (see Supplementary Note 1). In September 2012, a skeleton (Skeleton 1) was excavated at the presumed site of the Grey Friars friary in Leicester, the last-known resting place of Richard III (ref. 6). The archaeological, osteological and radiocarbon dating evidence were all consistent with the remains being those of Richard III (ref. 6). The skeleton was that of a male aged 30 to 34 years^7^, with severe scoliosis rendering one shoulder higher than the other^8^, with numerous perimortem battle injuries^7^. Modelled radiocarbon dating was also consistent (1456–1530AD at 95.4% probability) with these being the remains of an individual who died in 1485 (refs 6, 9). What has been missing to date is the genetic and genealogical data, and an integrative analysis of both the genetic and non-genetic lines of evidence. We therefore conducted ancient and modern DNA analysis, and, for the first time, a synthesis of all the evidence together, to come to an overall conclusion about the identity of Skeleton 1.

Analysis of the complete mitochondrial DNA (mtDNA) sequence from Skeleton 1 shows a perfect match with the mtDNA sequence of one living female-line relative of Richard III and a single substitution when compared with a second living female-line relative. The Y-chromosome haplotype from Skeleton 1 does not match that of male-line relatives of Richard III, but this is not remarkable given that a false-paternity event could have occurred in any of the intervening generations. While no contemporary portraits of Richard III survive, the DNA-predicted hair and eye colour are consistent with Richard’s appearance in one early portrait. Finally, an integrative Bayesian analysis results in a conservative overall likelihood ratio of 6.7 million, showing beyond reasonable doubt that Skeleton 1 is the remains of King Richard III.

## Results

### Sex determination of the remains

The sex of the remains was determined by amplification of segments of the X- and Y-chromosomes (see Supplementary Methods and Supplementary Tables 1 and 2)^10^. The results confirmed the remains being those of a male individual, as also suggested by physical analysis of the bones.

**Figure 1:**
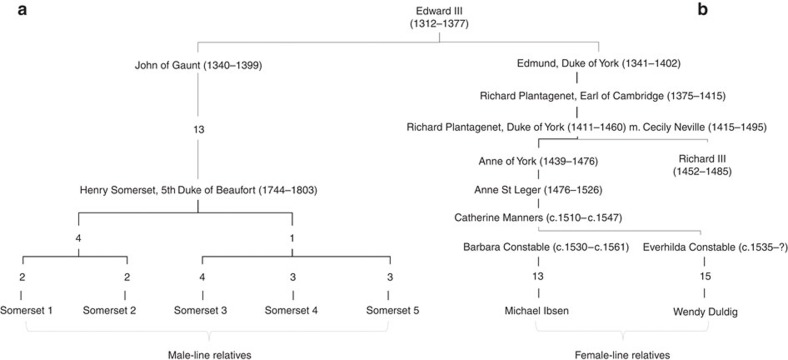
Genealogical links between Richard III and modern-day relatives who participated in this study. (**a**) Genealogical information links the Somersets to Richard III through an all-male line (left-hand side) through Edward III. Numbers indicate the number of individuals in the tree between named individuals. Two illegitimacy events where sons born out of wedlock were later legitimized are known to have occurred in the period between John of Gaunt and Henry Somerset, 5th Duke of Beaufort. (**b**) Genealogical information links Michael Ibsen and Wendy Duldig to Richard III through a female-only line (right-hand side) descended from Richard III’s eldest sister, Anne of York. Numbers indicate number of individuals in the tree between named individuals.

DNA identification beyond simply determining the sex of the remains relies on comparison with known relatives. One of the key problems with deep historical relatedness is that, for recombining portions of the genome, the sharing of DNA segments between relatives decays rapidly with the number of generations separating them. Therefore, after several generations, only the uniparentally inherited mitochondrial genome and non-recombining part of the Y-chromosome can be informative about relatedness. As such, only individuals matrilinearly or patrilinearly related to Richard III would be useful for comparison. As Richard III left no living descendants, it was necessary to find individuals related to him through other genealogical links (see Fig. 1, Supplementary Figs 1 and 2 and Supplementary Note 2).

### Male-line relatives and Y-chromosome analysis

Male-line relatives are generally easier to trace than female ones historically, and ennobled and titled lineages are recorded in a number of published sources^11^. We were able to identify, locate and contact five such relatives, descended from the 5th Duke of Beaufort (1744–1803), who agreed to take part in the study, providing an, albeit distant (between 24 and 26 generations), set of patrilinear relatives (see Fig. 1a and Supplementary Fig. 2). It is worth noting that while easier to trace genealogically, the male line is far more susceptible to false-paternity than the female line is to false-maternity events^12^.

Four of the modern relatives were found to belong to Y-haplogroup R1b-U152 (x L2, Z36, Z56, M160, M126 and Z192)^13^,^14^ with STR haplotypes being consistent with them comprising a single patrilinear group. One individual (Somerset 3) was found to belong to haplogroup I-M170 (x M253, M223) and therefore could not be a patrilinear relative of the other four within the time span considered, indicating that a false-paternity event had occurred within the last four generations.

Sequencing of Y-chromosome single-nucleotide polymorphisms (SNPs) on Skeleton 1 was carried out by on-array DNA hybridization capture^15^ of 24 amplified Illumina^16^ sequencing libraries, using probes generated to cover SNPs relevant to the major European Y lineages, followed by sequencing on a single 100 SE Illumina Hiseq 2000 sequencing lane. This approach provided insufficient coverage for some SNPs and further typing was performed using targeted PCRs with the amplification products sequenced on an Ion Torrent PGM. Finally, we also generated a STR haplotype using the Promega PowerPlex Y23 system (see Supplementary Figs 3 and 4 and Supplementary Tables 3–5).

In contrast to the Y-haplotypes of the putative modern relatives, Skeleton 1 belongs to haplogroup G-P287, with a corresponding Y-STR haplotype. Thus, the putative modern patrilinear relatives of Richard III are not genetically related to Skeleton 1 through the male line over the time period considered. However, this is not surprising, given an estimated average false-paternity rate of ~1–2% (refs 12, 17, 18). The putative modern relatives and Richard III are related through a male relative (Edward III) four generations up from Richard III (Fig. 1a and Supplementary Fig. 2), and a false-paternity event could have happened in any of the 19 generations separating Richard III and the 5th Duke of Beaufort, on either branch of the genealogy descending from Edward III. Indeed, even with a conservative false-paternity rate^18^ (see Supplementary Methods) the chance of a false-paternity occuring in this number of generations is 16%.

### Female-line relatives and mtDNA analysis

In contrast to false-paternity, false-maternity is, for obvious reasons, much less likely. However, historical records of female-line lineages are usually more difficult to track over multiple generations due to the change of surname on marriage. Fortunately, the family trees of noble families and other landed elites are often better recorded and a family tree showing an unbroken female lineage tracing from Anne of York, Richard’s eldest sister, down to the early 19th century was published in a number of sources^19^ and a modern descendant family identified^20^,^21^. However, as no supporting evidence or documents for these identifications were reported, we carried out additional genealogical research to fully document this first lineage and, furthermore, traced a second female lineage (see Supplementary Notes 2 and 3). Thus, we were able to obtain samples for comparison from Michael Ibsen (ML1), 19 generations removed from Richard III on the female line, and from Wendy Duldig (ML2), 21 generations removed from Richard III (see Fig. 1b and Supplementary Fig. 1). Wendy Duldig and Michael Ibsen are female-line 14th cousins, twice removed (32 meioses). Furthermore, we undertook a reconstruction of Richard’s kinship network at the time of Bosworth to eliminate, as far as possible, known contemporary relatives sharing a common inherited mtDNA type (see Supplementary Note 2b).

We carried out mtDNA analyses in two stages. In the first stage, both strands of the mtDNA control region (1,210 bp) were sequenced in duplicate from both ML1 and ML2 using Sanger sequencing. No sequence differences were observed between either duplicated samples from the same individual or individuals. Three hypervariable sections (HV1, HV2, HV3) (ref. 22) of the mtDNA control region of Skeleton 1 were sequenced from two independent extractions carried out in two different ancient DNA laboratories. Sanger sequencing of cloned PCR products was also performed and no sequence differences were observed except for two that can be attributed to DNA damage patterns found in ancient DNA^23^,^24^,^25^. We found a perfect match between all three individuals (ML1, ML2 and Skeleton 1), consistent with these individuals all being matrilinear relatives at the genealogical time depth considered.

To determine the full mtDNA similarity, we carried out complete mitochondrial genome sequencing on all three samples. For the modern samples (ML1 and ML2), the entire mitochondrial genome was amplified via two long-range PCRs^26^ in duplicate, followed by sequencing on an Ion Torrent PGM: all sites differing from the revised Cambridge reference sequence (rCRS)^27^ were subsequently confirmed by Sanger sequencing on both strands in both ML1 and ML2 in duplicate.

Whole-genome sequencing of the mtDNA sequence of Skeleton 1 was carried out using on-array DNA hybridization capture^15^,^16^ on 24 sequencing libraries generated from 16 extracts using probes generated from the mtDNA sequence of the two modern relatives followed by sequencing on a single 100 SE Illumina Hiseq 2000 sequencing lane (see Supplementary Fig. 5). These revealed a perfect whole-genome sequence match with ML1 and a single difference (position 8,994) with ML2 consistent with these individuals being matrilinear relatives over the time period considered^28^ (see Supplementary Tables 6–8).

Next we investigated the probability that the mtDNA match between Skeleton 1 and ML1 could have occurred by chance. No matches with the observed sequence were found in a database of 26,127 European complete mtDNA control region sequences (http://empop.org/)^29^ nor in a database of 1,832 British Isles samples^30^ covering only positions 16,093–16,320 and 00073–00188, thereby suggesting that the haplotype is rare. MtDNA haplotype frequencies do not vary greatly across Europe (http://empop.org/) and female mobility among the European nobility tends to be higher than the general population. Therefore, the absence of any match among the 26,127 European sequences would justify a conservative match probability value of around 1 in 10,000. However, to err on the side of caution, we used only the smaller, lower-resolution British database to obtain a very conservative match probability value of 2/1,832 (see Supplementary Methods).

### DNA-predicted hair and eye colour

Genetic data can also be used to infer phenotypic traits such as hair and eye colour^31^,^32^. There are no contemporary portraits of Richard III (ref. 33), all of them post-dating his death by some 25 years or more (see Supplementary Note 4 and Supplementary Fig. 6). Dendrochronological analysis has confirmed that the earliest of all known portraits of Richard III to have survived are the Society of Antiquaries of London (SAL) Arched-Frame portrait and the portrait in the Royal Collection, both thought to date within a few years of each other in the 1510s. The SAL portrait is very different from other paintings of the king, which appear to derive from an original type represented by the portrait held by the Royal Collection. The SAL portrait also has not been subject to significant later overpainting^33^.

**Figure 2:**
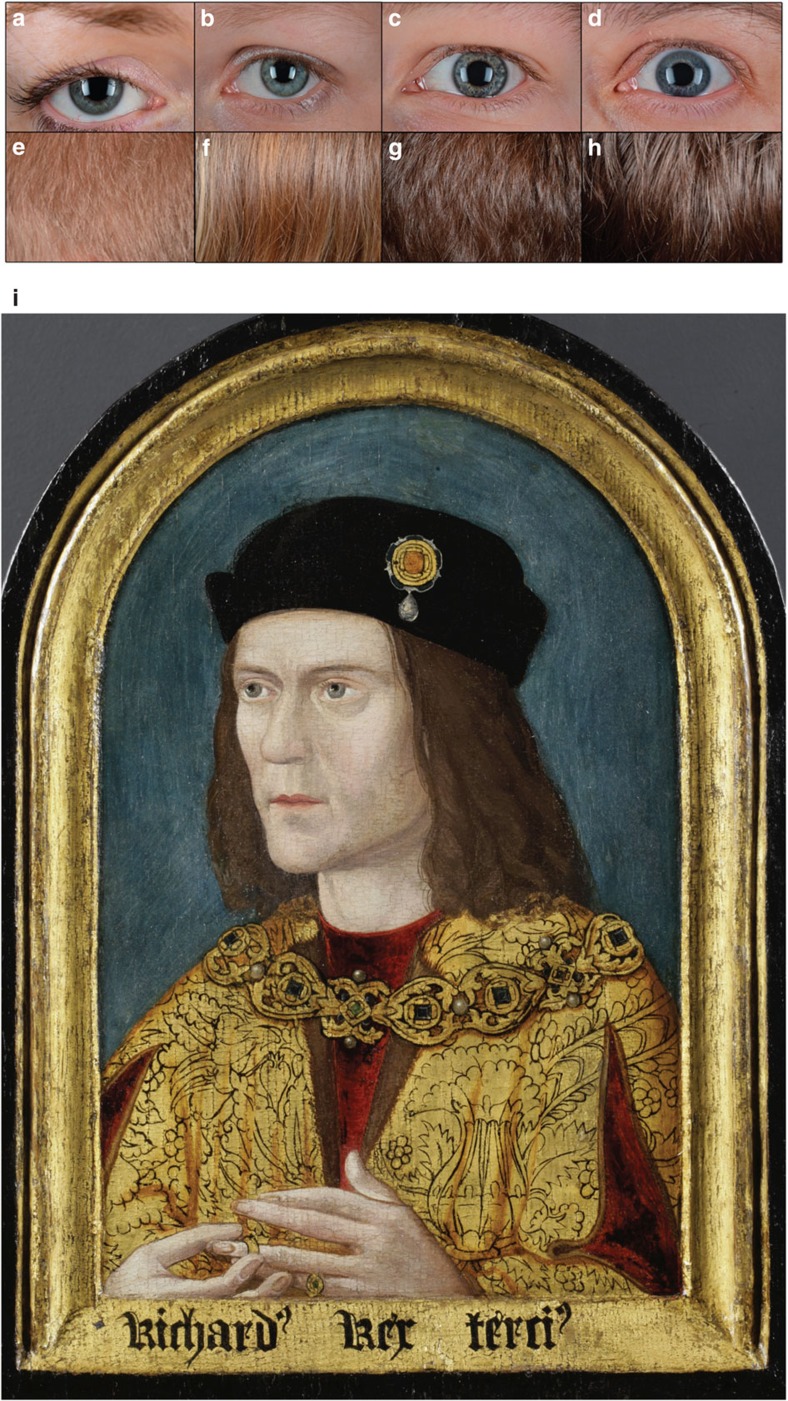
Hair and eye colour prediction from genetic data. Proposed eye and hair colour predictions for Richard III’s profile based on a set of individuals with similar eye and hair colour prediction probabilities. **a**–**d** give several examples that show the possible range of eye colours associated with a high blue probability of 0.955 using the IrisPlex model. **e**–**h** give several examples that show the possible range of hair colours associated with a high blond probability of 0.771 using the HIrisPlex model. It should be noted that eye and hair colour prediction has yet to reach individualized continuous colours. Therefore, these examples are provided as an indication of what colours may be possible using the 24-SNP HIrisPlex genotype profile. (**i**) The Society of Antiquaries of London Arched-Frame Portrait. Portrait reproduced with kind permission of the Society of Antiquaries of London.

Eye and hair colour DNA typing was carried out using probes designed for the HIrisPlex^31^ SNPs and, where necessary, followed by directed PCR using newly designed primers to generate amplicons under 100 bp in length, followed by sequencing on an Ion Torrent PGM (see Supplementary Table 9). Phenotype predictions were produced from the IrisPlex and HIrisplex^31^ statistical models^34^. These results show that Skeleton 1 had a 96% probability of having blue eyes together with a 77% probability of having blond hair (see Fig. 2a–b and Supplementary Note 4). Figure 2a–d shows blue eyes of contemporary Europeans whose DNA predictions fall within the range of high blue probability estimated from the Skeleton 1 profile. Similarly, Fig. 2e and f shows blond hair colour within the range of the high blond probability estimated from the Skeleton 1 profile. However, current hair colour DNA predictions resemble childhood hair colour and it is important to note that in certain blond individuals, hair colour can darken during adolescence. It is therefore possible that Skeleton 1 had brown hair as represented in Fig. 2g and h as seen in contemporary Europeans with a similarly high blond probability as obtained for Skeleton 1. The painting of Richard III that most closely matches the genetically predicted eye and hair colour results is the SAL Arch-Framed portrait (see Fig. 2i and Supplementary Note 5).

### Statistical analysis

To obtain a probability that Skeleton 1 is that of Richard III, we considered the non-genetic data (radiocarbon data^6^, estimated age at death, sex, presence of scoliosis^8^ and presence of perimortem wounds^7^) together with the genetic data (mtDNA and Y-chromosome). For each data type, we computed likelihoods for the observed data under hypothesis 1 (H1—that Skeleton 1 is Richard III) and under hypothesis 2 (H2—that Skeleton 1 is not Richard III). Although the mtDNA evidence favours H1, the Y-chromosome evidence provided limited evidence against H1, and our conservative analysis of the genetic evidence only gave moderate support for H1 (likelihood ratio, LR=79; see Supplementary Methods). Using a sceptical prior probability of 0.025 that skeleton 1 is that of Richard III, we obtained a posterior probability of 2/3 that H1 is true. On the other hand using a prior probability of 0.5, the genetic evidence lead to almost 99% probability that H1 is true. This analysis is highly conservative because, first, it used a low rate for false-paternity events, and second, the probability of a mtDNA match by chance (match probability) used was greater than 0.001, much higher than would be suggested by the absence of control region matches in the European database (*n*=26,127, LR=6,847). Furthermore, this analysis does not take into account the whole-genome mtDNA match with one modern relative and a single-base difference with a second. We note that if we ignore the Y-chromosome evidence, because of its susceptibility to false-paternity events, the contribution of the genetic data strengthens considerably (LR=478). The non-genetic evidence strongly supports H1 (LR=85,000). All the evidence combined is therefore extremely strong in supporting H1 (LR=6.7 million). This LR leads to a probability that H1 is true between 0.999994 (sceptical prior) and 0.9999999 (0.5 prior, see Supplementary Table 10). All likelihoods were computed under conservative assumptions (discussed in the Supplementary Methods) and therefore, these reported values are almost certainly lower than justified by the evidence.

## Discussion

The search for the remains of Richard III can be likened to a missing person’s case, with such investigations becoming more difficult the longer the time between the investigation and the time of death of the individual^35^,^36^. Given the 527 years that had elapsed since Richard’s death at Bosworth, this case is of special interest in that it is the oldest DNA identification case of a known individual to date. As with any such case, all quantitative strands of evidence should be drawn on to reach a conclusion regarding the identity of any putative candidate. This report is the first that draws all such available strands together and estimates the statistical support for the skeletal remains discovered in 2012 being those of the last Plantagenet king, Richard III.

In drawing the evidence together, historical documents indicated that we would be looking for the remains of an individual who was described, during his lifetime, as having one shoulder higher than the other, who, in 1485, aged 32, died in the heat of battle before being brought back to Leicester to be buried in the choir of the church of the Grey Friars. In September 2012, the remains of an individual fitting all these criteria were found. Subsequently, in addition to the compelling archaeological evidence, laboratory analyses provided information on radiocarbon dating^6^, isotopic analyses^9^, the degree and nature of the scoliosis^8^ as well as the injuries sustained^7^. We present the genetic analysis of the remains and the only known female-line relatives of Richard III and find a positive mtDNA match. Whilst there was no Y-chromosome match between the skeletal remains and five genealogically determined male-line relatives, given the known possibility of a false-paternity over several generations, this did not prove to be a highly significant factor. One can speculate that a false-paternity event (or events) at some point(s) in this genealogy could be of key historical significance, particularly if it occurred in the five generations between John of Gaunt (1340–1399) and Richard III (see Supplementary Fig. 2). A false-paternity between Edward III (1312–1377) and John would mean that John’s son, Henry IV (1367–1413), and Henry’s direct descendants (Henry V and Henry VI) would have had no legitimate claim to the crown. This would also hold true, indirectly, for the entire Tudor dynasty (Henry VII, Henry VIII, Edward VI, Mary I and Elizabeth I) since their claim to the crown also rested, in part, on their descent from John of Gaunt. The claim of the Tudor dynasty would also be brought into question if the false paternity occurred between John of Gaunt and his son, John Beaufort, Earl of Somerset. If the false paternity occurred in either of the three generations between Edward III and Richard, Duke of York, the father of Edward IV and Richard III, then neither of their claims to the crown would have been legitimate.

Analysing all the available evidence in a Bayesian framework, even using highly conservative measures, we conclude that the evidence is overwhelming that Skeleton 1 from the Grey Friars site in Leicester is that of Richard III, thereby closing a 500-year-plus missing person case.

## Methods

### Laboratory locations

All DNA work involving the modern relatives was carried out at the University of Leicester. Male-line relatives were typed using Promega PowerPlex Y23 and for SNPs defining the main European Y-haplogroups in Leicester with a subset of the typing being confirmed at the Université Paul Sabatier. Female-line relatives were sequenced for the entire mitochondrial genome at the University of Leicester. DNA was extracted from ancient teeth and bone at the University of York and the Université Paul Sabatier, Toulouse. Library preparation and target enrichment were done at the University of York. Single-end 100-bp sequencing using a HiSeq 2000 (Illumina, CA, USA) was performed at the Copenhagen Sequencing Facility. Targeted sequencing of both modern and ancient DNA was also carried out at genomic technical platform PlaGe (Genopole, Toulouse, France) and at the Protein Nucleic Acid Chemistry Laboratory at the University of Leicester. Below we provide a condensed version of the methods used. For each step, full information can be found in the Supplementary Methods. Details surrounding the extensive genealogical research carried out for this project can be found in the Supplementary Note 2.

### Sample collection

DNA was extracted from saliva samples of the modern relatives of Richard III and all participants were recruited with informed consent following project review by the University of Leicester Research Ethics Committee.

Skeleton 1 was excavated and samples taken under clean conditions^37^. Everyone involved in the excavation at the Grey Friars site, the clean laboratory in Leicester and those involved in the laboratories and labwork had their mitochondrial and, for males, Y-chromosomes typed. DNA was extracted from saliva samples and all participants were recruited with informed consent.

### DNA extraction of ancient samples

DNA was extracted from teeth and bone (femur) samples. All procedures were performed in dedicated ancient DNA laboratories at the University of York and the Université Paul Sabatier, Toulouse with appropriate contamination precautions in place. Two extraction blanks were included and treated exactly as if they were extracts throughout the whole process. PCRs and library experiments also included further blank controls.

### Sex-typing assay performed on Skeleton 1

A newly designed sex-typing assay comprising of PCR primers for co-amplification of the SRY fragment with UTX and UTY homologous regions was used. This assay was designed to enable relatively small sized fragments of SRY, UTX and UTY to be co-amplified from samples likely to contain degraded DNA^10^.

### Mitochondrial control region analysis of Skeleton 1

Analysis of the hypervariable segments (HV1, HV2 and HV3) of the mtDNA control region was carried out by amplifying and directly sequencing multiple overlapping fragments ranging from 153 to 250 bp in size (http://forensic.yonsei.ac.kr/protocol/mtDNA-midi-mini.pdf)^22^. A selection of amplicons was used for cloning the PCR products in the lab in Toulouse. Sequencing was carried out using the Big-Dye Terminator V3.1 cycle sequencing kit (Applied Biosystems) and by capillary electrophoresis on an ABI Prism 3730 Genetic Analyser (Applied Biosystems) at the Protein Nucleic Acid Chemistry Laboratory at the University of Leicester or at the genomic technical platform PlaGe (Genopole).

### Mitochondrial genome/Y-SNP/HIrisplex typing of Skeleton 1

Libraries were built following Meyer and Kircher^16^, with the exception that the first filtration step between the blunt end repair and the adapter ligation was substituted by heat inactivation of the enzymes^38^,^39^. Two microarrays were designed, one for the mtDNA enrichment and another one for nuclear SNP enrichment. DNA enrichment was performed by hybridization capture using the Agilent 244k DNA SureSelect microarray (Agilent, Böblingen, Germany). For the nuclear capture, Y-chromosome probes were designed to cover the SNPs relevant to the major European lineages^14^. Further probes were designed to cover the SNPs relevant to the HIrisplex^31^ markers. These two sets of probes (mitochondrial and SNPs) were used separately to fill the two different microarray designs of a 1 × 244K format. For each microarray, the capture protocol was performed following Hodges *et al.*^15^ with the modifications proposed by Zhang *et al.*^40^ and Fortes and Paijmans^38^. The libraries were pooled in equimolar quantities and sequenced on two lanes of the Illumina HiSeq 2000 platform in 100 SE mode at the sequencing facility of the University of Copenhagen, Denmark.

The raw reads from each library were sorted based on the six-nucleotide index used during library preparation. Only reads with a 100% match to the index were selected for further analyses. Reads shorter than 25 nucleotides were discarded from further analysis. The trimmed reads were mapped to autosomes and sex chromosomes from the human reference genome build 37 (GRCh37) and to the rCRS (NC_012920.1) using bwa 0.7.5a-r405 (ref. 41). In each alignment, the output bam files were merged using SAMtools 0.1.19 (ref. 41) and PCR duplicates were removed subsequently. The mapped reads were filtered based on a mapping quality >29 and their alignment to unique positions along the reference sequence.

Polymorphic positions were identified using SAMtools (SAMtools 0.1.19) and bcftools. Finally, vcfutils.pl was used to filter the list of variants according to a Phred-scaled genotype posterior probability quality >20 and a read depth higher than 10. To avoid miscalling because of the deamination pattern of ancient DNA molecules, all the polymorphic positions reported in the vcf output file were checked by eye. In the case of the mitochondrial genome, the assembly to the reference was visualized in Tablet^42^, while the alignment of the reads containing the SNPs to the reference chromosomes was visualized using IGV^43^.

### SNP typing by PCR

The capture approach yielded insufficient coverage for all HIrisPlex and Y-chromosome SNPs and therefore primers were designed to generate amplicons containing these SNPs as well as two SNPs, which further define Y-chromosome haplogroup G: M285 (G1) and P287 (G2) (ref. 14). These were amplified as part of multiplex reactions following Römpler *et al.*^44^ or singleplex reactions (using 40 cycles and with no secondary amplification) and sequenced on the Ion Torrent following library preparation using Ion PGM 200 Xpress Template Kit and PGM 200 Sequencing Kit. To increase coverage, singleplex PCR and sequencing of one marker (rs28777) was carried out according to Binladen *et al.*^45^

Typing of the haplogroup G defining SNPs (M201, M285 and P287) was repeated in Toulouse using singleplex PCRs. Sequencing of these PCR products was carried out using Big-Dye Terminator V3.1 cycle sequencing kit (Applied Biosystems) analysed by capillary electrophoresis on an ABI Prism 3730 Genetic Analyser (Applied Biosystems) at the genomic technical platform PlaGe (Genopole).

### Y-chromosomal haplotype analysis

Ancient and modern samples' Y-chromosomal haplotypes were obtained using the PowerPlex Y23 System (Promega) and analysed by capillary electrophoresis on an ABI Prism 3730 Genetic Analyser (Applied Biosystems) at the genomic technical platform PlaGe (Genopole) and on an ABI Prism 3130xl Genetic Analyser (Applied Biosystems) at the University of Leicester. For Skeleton 1, this was carried out on three separate extracts (RM2, LM1 and LM3) in two different ancient DNA laboratories (York and Toulouse). For the modern relatives, this was carried out on two different extracts in two different modern laboratories (Leicester and Toulouse).

### Y-chromosomal SNP analysis of modern samples

Following determination of the Y-haplotype for the modern male-line samples, the predicted haplogroup was determined using Whit Athey’s haplogroup predictor (http://www.hprg.com/hapest5/hapest5a/hapest5.htm?order=num). Binary markers covering these and related lineages were typed in two multiplexes by the SNaPshot minisequencing procedure (Applied Biosystems) and an ABI3130xl Genetic Analyzer (Applied Biosystems) followed by confirmation using Sanger sequencing. Somerset 3 was determined to be Hg I (M170+ M223−, M253−)^14^ derived, further confirmed by the lab in Toulouse. Somersets 1,2,4 and 5 were determined to be derived for R1b-U152. Somersets 1,2,4 and 5 were tested for SNPs subdividing this clade^13^ (Z56, M126, Z36, Z192, M160 and L2) using Sanger sequencing in both labs.

### Modern mtDNA analysis

Both samples were replicated twice.

Samples were taken using Oragene DNA Collection kits (DNA Genotek) and DNA extracted using two different methods: the Qiacube Blood and Body Fluid protocol (200 μl with 200 μl elution) and the Oragene protocol. To analyse the control region, samples were sequenced twice in both the forward and reverse direction using two overlapping primer sets (15973-296 and 16524-614) using Big-Dye Terminator V 3.1 (Applied Biosystems). No differences were found between replicates or between samples.

Samples were amplified for the complete mitochondrial genome from both extractions following Meyer *et al.*^26^ PCR amplicons were sequenced on an Ion Torrent PGM Sequencer on an Ion314 Chip. Libraries were prepared using the Ion Xpress Plus gDNA Fragment Library Preparation kit, while the template preparation and the sequencing were carried out using the Ion PGM 200 Xpress Template Kit and the Ion PGM 200 Sequencing Kit, respectively. Raw reads were mapped back to the rCRS (NC_012920.1) using TMAP software included in the Ion Alignment plugin 3.2.1 (Torrent Suite Software 3.2.1) on the Ion Torrent server. Duplicate reads removal and variant calling were performed using SAMtools 0.1.19 (ref. 41) and local realigning was carried out with the Genome Analysis Tool Kit^46^. Variant sites were filtered for Base Quality 20, Mapping Quality 50 and Depth of Coverage 30 following which 33 polymorphic sites were retained. All these sites have been manually checked and confirmed by Sanger sequencing in both directions and replicated twice.

### Contamination control and quantification

Modern DNA contamination of the ancient remains was controlled for by the following methods:

1. Excavation was carried out under clean conditions (see Supplementary Methods)
2. Samples were stored in clean labs and ancient DNA work carried out only in dedicated ancient DNA facilities.
3. Separate ancient samples were processed in separate labs to replicate results.
4. All lab members and excavation participants had their mtDNA typed and Y-chromosome typing was carried out on all men involved. None had a matching mtDNA or Y-chromosome type.

As evidence against significant contamination, DNA analysis of Skeleton 1 shows a perfect mtDNA match to ML1 and a single-base difference with ML2. It also shows a clear Y-STR haplotype, which has been replicated using a number of extracts generated and tested in two separate labs. Finally, an examination (see Supplementary Methods) of the substitution pattern in our reads also supports this.

### Statistical analysis

Taking a conservative approach at each step, we computed a likelihood for each item of observed evidence under each of two opposing hypotheses: Hypothesis 1 (H1): Skeleton 1 is Richard III, and Hypothesis 2 (H2): Skeleton 1 is not Richard III.

As it was reasonable to assume that all the different lines of evidence were independent, the joint likelihood of all the evidence was obtained by multiplication of the individual likelihoods under each hypothesis. The weight of evidence for H1, called the likelihood ratio (LR), was then given by the ratio of the likelihood under H1 to that under H2. We say that an assumption is ‘conservative’ if it reduces the LR.

The LR can be converted into a probability that H1 is true, given a prior probability. We took as starting point the moment that Skeleton 1 was first observed and recognized as a human skeleton, but before any assessments of age, sex, state of health and cause of death were made. At that point, there was substantial evidence that a skeleton found in what is believed to have been the location of Leicester Grey Friars choir could be that of Richard III. All of the information available at the time that Skeleton 1 was unearthed, including its precise location and the nature of the grave, was regarded for this analysis as background information that can inform the prior probability. On the basis of that information, we believe that a sceptical observer could not reasonably have assigned a prior probability less than 1 in 40. This value was proposed in a previous analysis (http://rationalgareth.com/), based on what we judge to be sceptical assessments. The highest probability that could be justified by the prior evidence might be 1 in 2.

We have used relevant, available data where possible. Inevitably subjective judgments are required, for example, the relevant reference populations and about the probabilities of error in reported facts. As far as seemed possible and reasonable, we strived to be conservative in our approach, for example, using a pseudocount method to bias the LR towards a neutral value of 1, thus tending to avoid spurious large values from low observed frequencies. Details of the data and methods used in the statistical analysis of the radiocarbon data, age and sex of skeleton, presence of scoliosis, presence of perimortem wounds, Y-chromosome and mtDNA frequency data can be found in the Supplementary Methods. To summarize the results: the radiocarbon data yielded a likelihood ratio of 1.84 representing limited support for H1. The age and sex data yielded a likelihood ration of 5.25, again representing limited support for H1. The presence of one shoulder higher than the other, reported during Richard’s lifetime, could be attributed to scoliosis (Skeleton 1 had severe idiopathic adolescent-onset scoliosis) or two other known conditions, Erb’s Palsy and Sprengel’s deformity, both of which are very rare. Under H1, the above rates give an estimated probability of 0.90 of observing scoliosis given the description of Richard III’s physical appearance (=the scoliosis rate divided by the sum of the three rates), which we multiplied by 0.95 to allow for the possibility that the recorded description was incorrect. This lead to a LR of 212, providing moderately strong support for H1. The presence of perimortem injuries gave a LR of 42, and so moderate support for H1. The Y-chromosome of Skeleton 1 did not match that of genealogically determined patrilineal relatives of Richard III. This could be explained by a false-paternity event in one or more of the 19 putative father–son links between Richard III and Henry Somerset, fifth Duke of Beaufort. The Y-chromosome results also indicate one further false-paternity event between Henry Somerset and his five contemporary, presumed patrilinear descendants. To be conservative, we selected a published false paternity rate that was (1) lower than any other published rate that we considered^17^,^47^ and (2) based on genealogical data^18^. To this we add the false-paternity event in the 19 putative father–son links between Henry Somerset and five contemporary male Somersets. This gives a probability of at least one false paternity event in the 19 putative father–son links between Richard III and Henry Somerset of 0.16. Given that a false-paternity event must have occurred under H1, the population frequency of Skeleton 1’s Y-haplotype is the same under H1 and H2 and cancels out in the LR calculation. Thus, the LR is 0.16, representing limited evidence against H1.

The mtDNA sequences of Skeleton 1 and the presumed 19-meiosis matrilinear relative of Richard III, Michael Ibsen, matched completely. A 21-meiosis relative also matched except at one base (8994). The latter observation is equally likely under H1 and H2 given the observed sequence of Michael Ibsen, and so cancels out in the LR. Thus, we only need likelihoods for the observation of the sequence shared by Michael Ibsen and Skeleton 1.

To obtain the likelihood under H1, we require the mtDNA mutation rate, and in this case high estimates are conservative. Parsons *et al.*^28^ report 10 control region mutations in 327 generations using genealogical data. Because this suggests a higher rate than other published estimates, and is based on genealogical data, we used it to derive a probability of 0.52 for no mutation in 19 meioses.

For the likelihood under H2, we require the population fraction of the Skeleton 1 haplotype. Although we obtained the complete mtDNA genome sequence from Skeleton 1, we identified little published whole-genome comparison data from England. Thus, for the statistical analysis, we used only the mtDNA control regions between positions 16,093 and 16,320 and between 00073 and 00188, for which we obtained suitable English comparison data from an update of Röhl *et al.*^30^, supplemented with mtDNA sequences supplied by Roots for Real (Genetic Ancestor Ltd., Clare, Suffolk, UK). Using only these short sections of the control region under H2 is conservative, since the population fraction of the observed control region sequences cannot be less than that of the full mtDNA genome. The relevant reference population is over 500 years in the past, but due to the large population size over the period considered, we expect population frequencies to have changed little over the last five centuries. We found the frequency of the Skeleton 1 haplotype to be 0 among 1823 in the database, to which we add the one instance observed in Michael Ibsen. This approach is, again, conservative as Michael was sampled due to his known genealogical relationship to Richard III. This leads to an LR of 478 representing moderately strong evidence for H1.

We also noted that there were no matches in a database of 26,127 European mitochondrial control region haplotypes (www.empop.org)^29^. We do not rely on this database because it is Europe-wide rather than specific to England and because of ascertainment issues, but it suggests that the Skeleton 1 haplotype may be much rarer than can be inferred from our smaller English database. We also note that female mobility among the European nobility is likely to have been much higher than for the general population, because of marriage practices relating to political alliance formation. Such practices would provide some justification for using the European mtDNA database, and so for considering the haplotype found in Skeleton 1 and Michael Ibsen to be extremely rare. In Supplementary Table 10, we show some illustrative results using the European database to demonstrate the implications of establishing that the Skeleton 1 haplotype is as rare as suggested by that database.

The LRs for different combinations of the evidence, and two posterior probabilities, are shown in Supplementary Table 10. Using all the evidence, the support for H1 is extremely strong with an LR of 6.7 million, so that our sceptic would be driven to the conclusion that the probability that Skeleton 1 is not Richard III is less than 1 in 100,000, while for those taking a 1 in 2 starting position that probability is much less than 1 in a million. Taking into account the conservative assumptions underlying our calculation described above, we regard this as establishing the truth of H1 beyond reasonable doubt.

## Author contributions

T.E.K., M.H. and K.S. led the project. Ancient sample: T.E.K. and J.A. carried out the excavation and sample collection. Samples were stored in the Space Research Centre at the University of Leicester. J.H. provided space for storage, maintained and monitored storage conditions within his clean lab. Ancient DNA extractions were performed by T.E.K., G.G.F. and L.T. PCR amplifications on ancient DNA were carried out by T.E.K. and L.T. Library construction and capture was carried out by T.E.K. and G.G.F. Probes were designed by T.E.K. and G.G.F. Y-chromosome PCR primers were designed by T.E.K., M.K. and P.B. Analysis of data (mapping, SNP calling, mtDNA assembly, Y-chromosome analysis) was carried out by T.E.K., G.G.F., P.B., P.M.D., M.H., W.P. and R.N. Modern samples: sample collection and DNA extraction was carried out by T.E.K. PCRs were carried out by T.E.K. and L.T. Next-generation sequencing of the mtDNA was carried out using the Ion Torrent PGM by T.E.K. and R.N. Sequencing of Y-SNPs was carried out by T.E.K., R.N. and L.T. Analysis of mitochondrial data was carried by T.E.K., R.N. and P.M.D. Sequencing and analysis of Y-chromosome data was carried out by T.E.K., P.B., R.N. and P.M.D. Data on mtDNA types and mutation rates were provided by W.P., M.H. and P.F. Prediction of hair and eye colour based on HIrisplex allele calls was carried out by S.W. and M.K. Statistical analysis was carried out by M.G.T., T.E.K. and D.B., D.E. provided information and expertise on the portraits. Genealogical research for this project was led by K.S., T.K., K.S. and M.H. wrote the majority of the manuscript with critical input from G.F., P.B., W.P., P.M.D., M.T., D.B. and all other authors.

## Additional information

**How to cite this article:** King, T. E. *et al.* Identification of the remains of King Richard III. *Nat. Commun.* 5:5631 doi: 10.1038/ncomms6631 (2014).

**Accession codes:** The mitochondrial DNA sequences generated in this study have been deposited in GenBank under the accession codes KM676292 to KM676294.

## Supplementary Material

Supplementary Information Supplementary Figures 1-6, Supplementary Tables 1-10, Supplementary Notes 1-5, Supplementary Methods and Supplementary References.
