# Supplementary material for: Identification of the remains of King Richard III
Source: Nat Commun. 2014 Dec 2;5:5631. doi: 10.1038/ncomms6631 (PMC4268703; doi:10.1038/ncomms6631)
Supplement: Supplementary Information — Supplementary Figures 1-6, Supplementary Tables 1-10, Supplementary Notes 1-5, Supplementary Methods and Supplementary References. [file ncomms6631-s1.pdf]

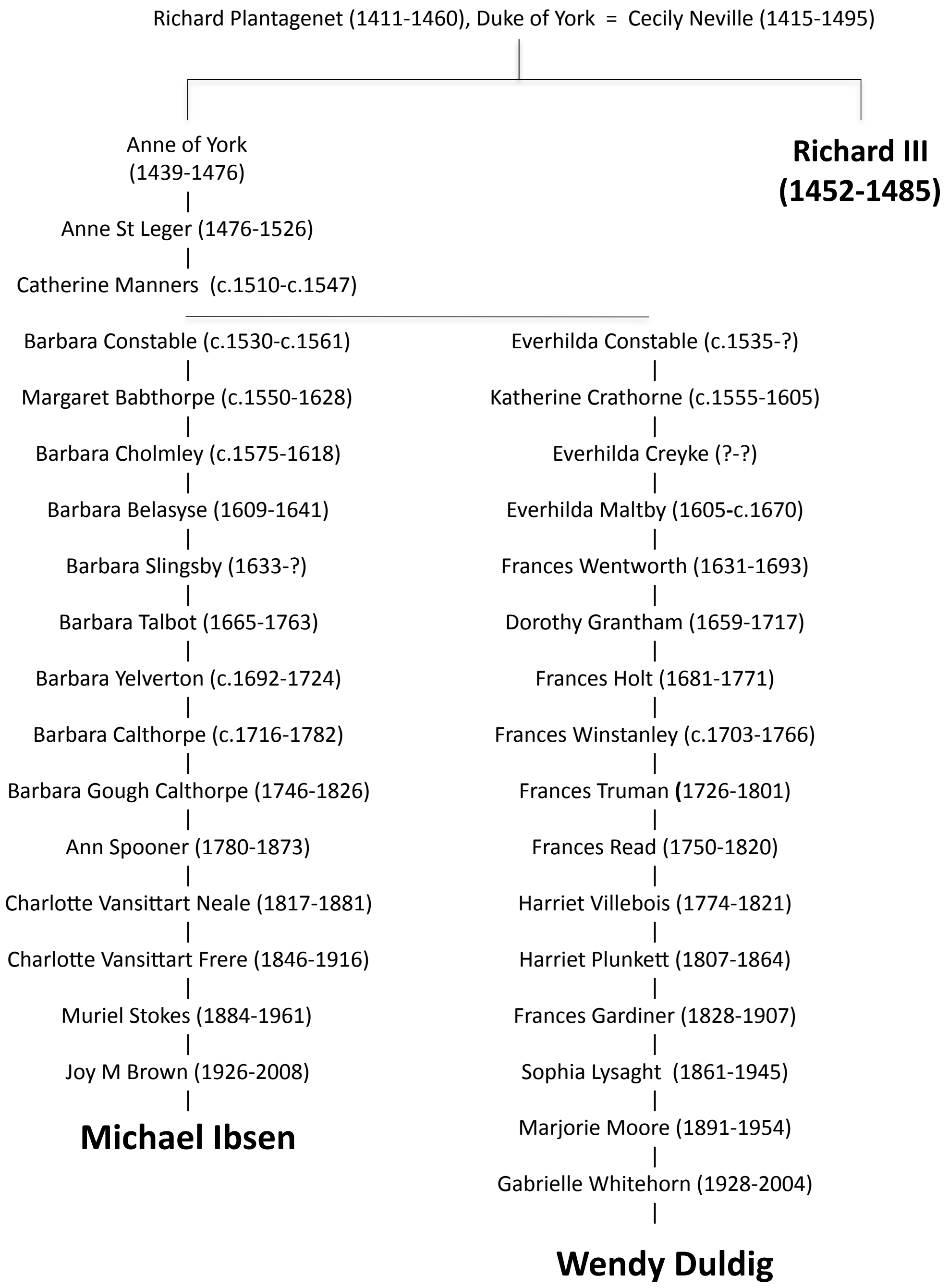

Supplementary Figure 1: Detailed family tree of the Ibsen and Duldig lineages

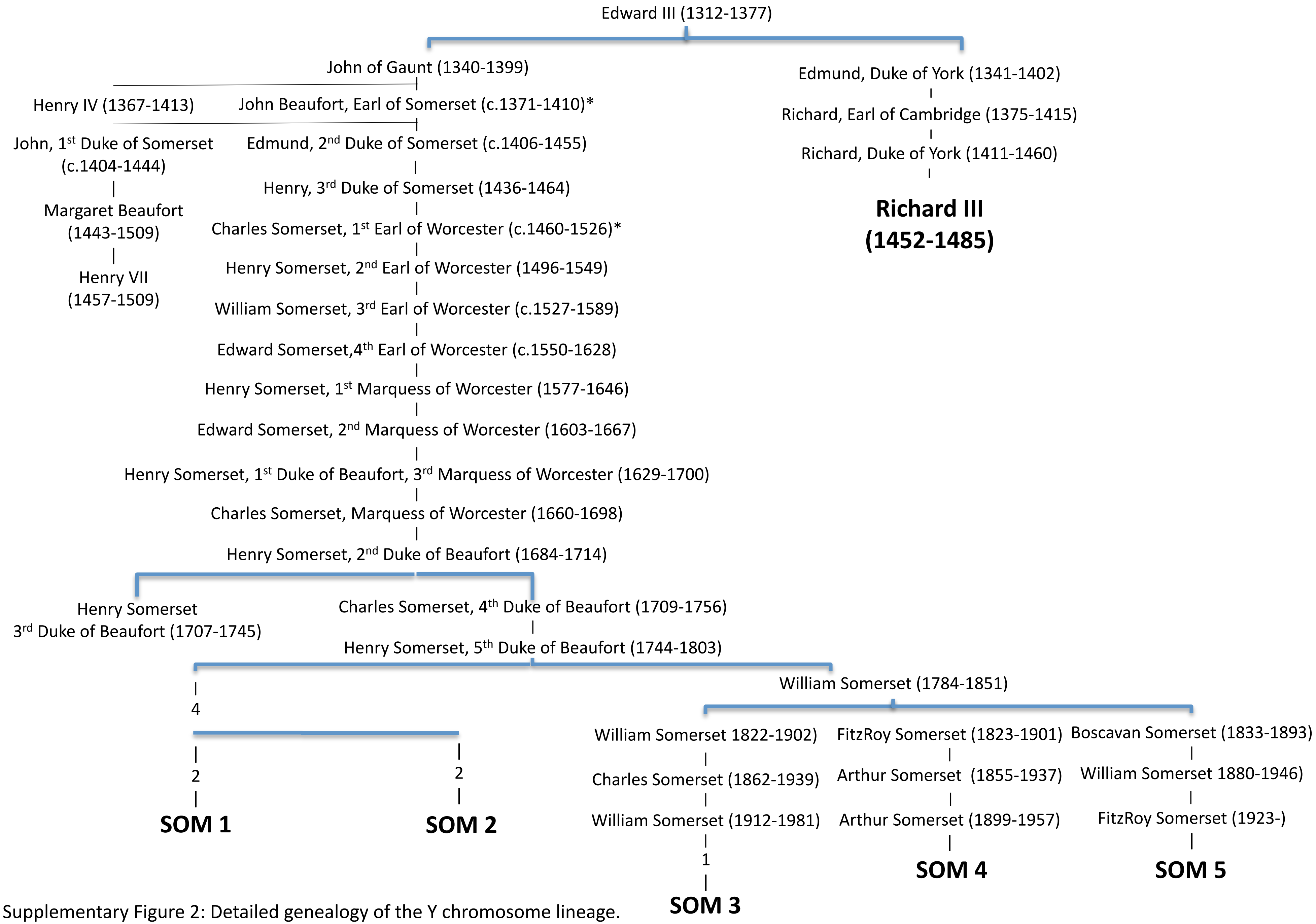

Supplementary Figure 2: Detailed genealogy of the Y chromosome lineage.
Numbers indicated number of individuals in genealogy between named individuals.
* indicates known occurrences of sons being born illegitimate and later legitimized

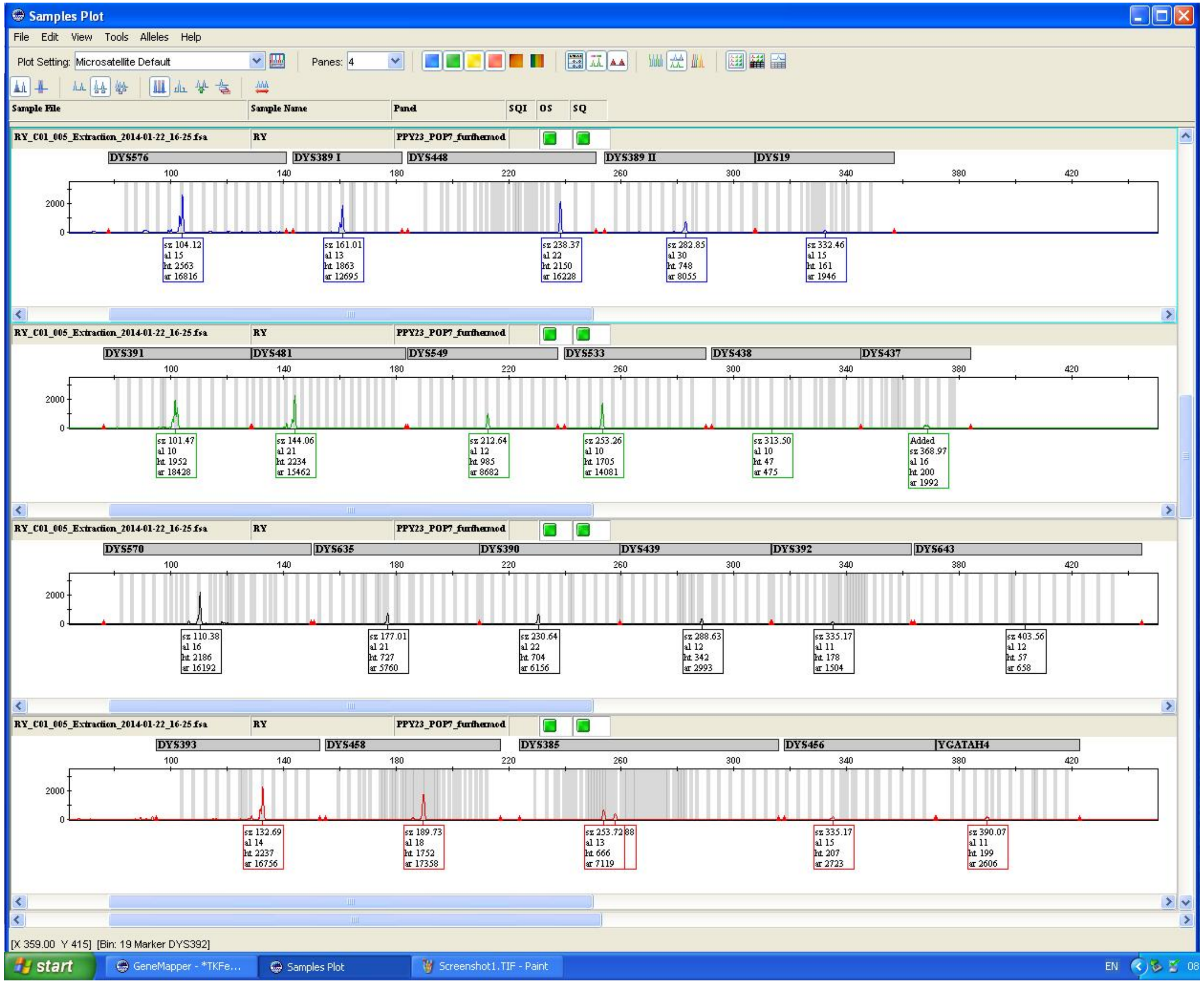

Supplementary Figure 3: Screenshot of Y-STR haplotype for Skeleton 1

Y chromsome haplogroup information

(as per Karafet et. al. 2008[36], Myres et. Al. 2010[37] and Rocca et al. 2013[48])

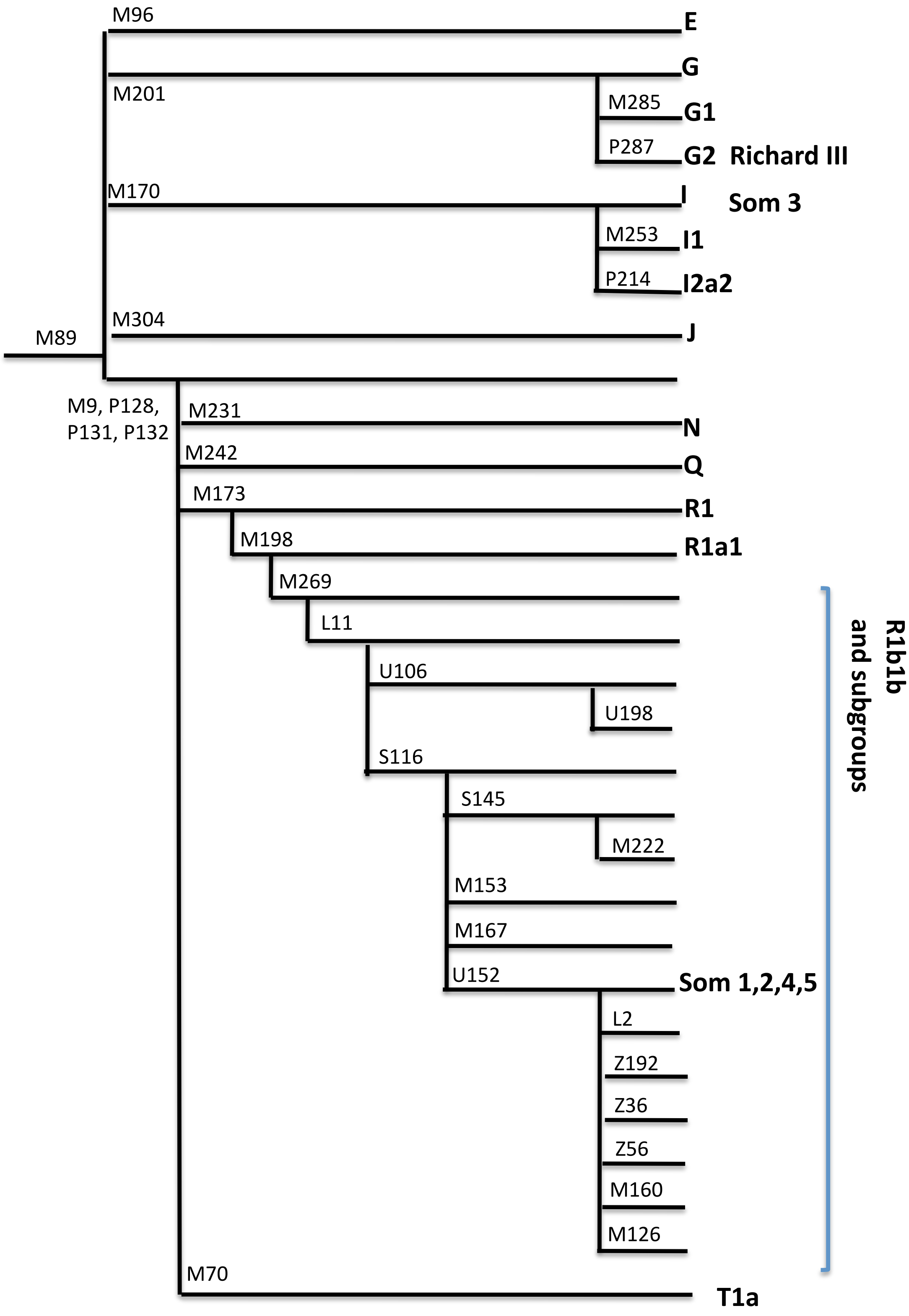

Supplementary Figure 4: Tree of Y chromosomal SNPs typed

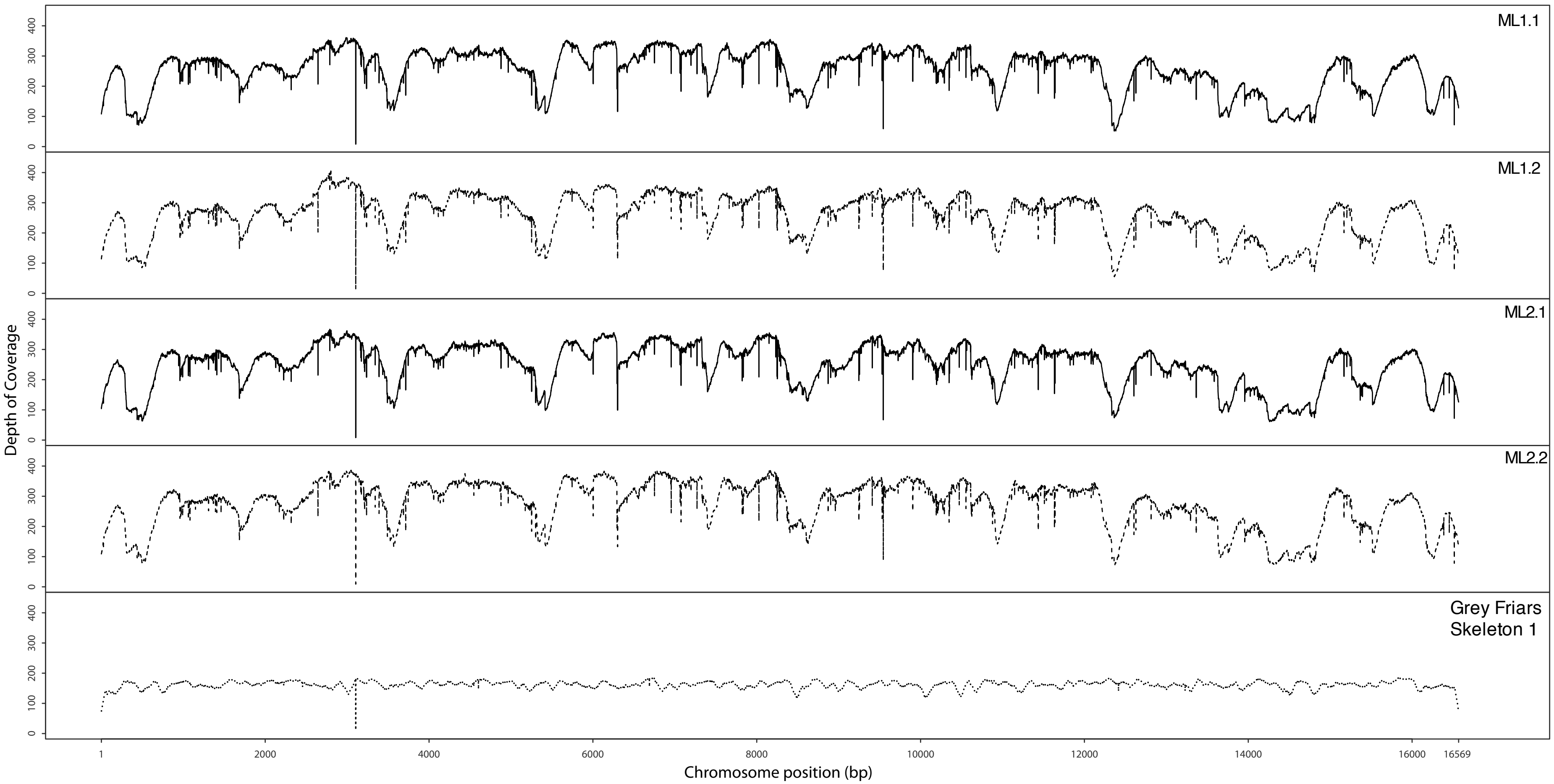

Supplementary Figure 5: Graphs of depth of coverage for the mitochondrial sequences. ML1.1 and ML1.2 represent the two replicates of ML1. ML2.1 and ML2.2 represent the two replicates of ML2.

King Richard III, The National Portrait Gallery, London
Late 16th century (late 15th century)

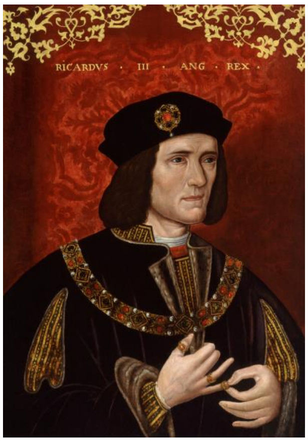

Image reproduced with kind permission of The National Portrait Gallery. ©The National Portrait Gallery, London.

King Richard III, The National Trust, Anglesey Abbey
(1500-1599)

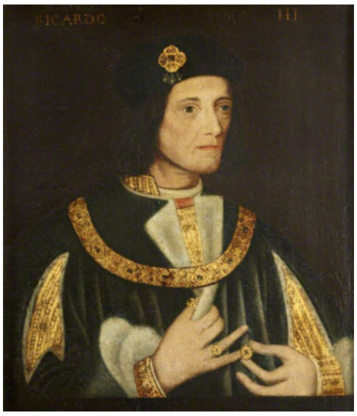

Image reproduced with kind permission of the The National Trust. The Fairhaven Collection (National Trust) © National Trust

King Richard III, The National Portrait Gallery, London
1590-1610

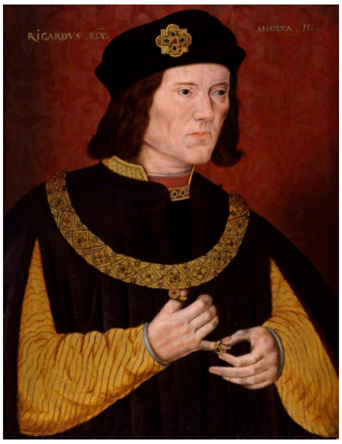

Image reproduced with kind permission of The National Portrait Gallery. ©The National Portrait Gallery, London.

King Richard III, Hatfield House
At Hatfield since 1611/12 at least, according to Hatfield House records

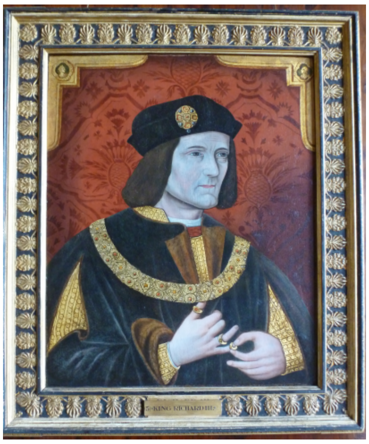

Image reproduced with kind permission of the Marquess of Salisbury, Hatfield House, Hertfordshire.

Supplementary Figure 6: Portraits of Richard III, all painted within ~120 years of his death, all of which appear to be copies of the portrait in the Royal Collection.

Supplementary Table 1: DNA extractions and libraries.

Source	Extraction no.	Amount used	Number of libraries	Capture
Right molar2	Extraction 1	420mg	0	no
	Extraction 2	300mg	0	no
Bone	Extraction 1	231mg	1	mtDNA
Left molar 1- 1 root only	Extraction 1	176mg	1	mtDNA
Left molar 1 - remainder of tooth	Extraction 1	250mg	1	mtDNA, Y chromosome and Hirisplex
	Extraction 2	250mg	1	mtDNA, Y chromosome and Hirisplex
	Extraction 3	250mg	1	mtDNA, Y chromosome and Hirisplex
	Extraction 4	250mg	1	mtDNA, Y chromosome and Hirisplex
Left molar 2 Root	Extraction 1	250mg	2	mtDNA, Y chromosome and Hirisplex
	Extraction 2	200mg	2	mtDNA, Y chromosome and Hirisplex
Left molar 2 Crown	Extraction 1	250mg	2	mtDNA, Y chromosome and Hirisplex
	Extraction 2	200mg	2	mtDNA, Y chromosome and Hirisplex
Left molar 3 Root	Extraction 1	250mg	2	mtDNA, Y chromosome and Hirisplex
	Extraction 2	250mg	2	mtDNA, Y chromosome and Hirisplex
	Extraction 3	213mg	1	mtDNA, Y chromosome and Hirisplex
Left molar 3 Crown	Extraction 1	250mg	2	mtDNA, Y chromosome and Hirisplex
	Extraction 2	250mg	2	mtDNA, Y chromosome and Hirisplex
	Extraction 3	250mg	1	mtDNA, Y chromosome and Hirisplex
			24 total	

Supplementary Table 2: Sex-typing assay (primers and PCR conditions).

Marker	Dye	Primer F			Primer R			Amplicon size in human ref (bp)
		**Sequence (5'-3')**	**Tm (°C)**	**[µM]°**	**Sequence (5'-3')**	**Tm (°C)**	**[µM]°**	
SRY	FAM	gcgaaactcagagatcagcaag	60.1	0.08	tgtgcctcctggaagaatgg	61.9	0,08	81
UTY	FAM	cagtgttaccagccttaacag	53.7	0.2	ggcaggtctacttttgtgag	52	0,1	91
UTX	FAM				tctgtggactaggtttgtggt	55.6	0,11	120

Supplementary Table 3: Results of Y-chromosome typing. R1b-U152* = R1b-U152 (xM160, M126, L2, Z192, Z36, Z56).

Sample Name	DYS19	DYS385	DYS385II	DYS389 I	DYS389 II	DYS390	DYS391	DYS392	DYS393	DYS437	DYS438	DYS439	DYS448	DYS456	DYS458	DYS635	YGATAH4	DYS481	DYS533	DYS549	DYS570	DYS576	DYS643	Haplogroup
Som1	14	11	14	13	29	23	11	13	13	14	12	12	20	15	18	23	12	22	12	13	19	19	10	R1b-U152*
Som2	14	11	14	13	29	23	11	13	13	14	12	12	20	15	18	23	12	22	12	13	19	18	10	R1b-U152*
Som4	14	11	14	13	29	23	11	13	13	14	12	12	20	15	18	23	12	22	12	13	18	19	10	R1b-U152*
Som5	14	11	14	13	29	23	11	13	13	14	12	12	20	15	18	23	12	22	12	13	18	19	10	R1b-U152*
Som3	15	12	15	14	30	23	10	11	13	14	10	11	18	14	20	21	10	26	13	12	17	17	12	I-M170 (xM223, M253)
Sk1	15	13	14	13	30	22	10	11	14	16	10	12	22	15	18	21	11	21	10	12	16	15	12?	G2-P287

Supplementary Table 4: Y-STR haplotyping of Skeleton 1.

Sample	DYS19	DYS385	DYS385II	DYS389 I	DYS389 II	DYS390	DYS391	DYS392	DYS393	DYS437	DYS438	DYS439	DYS448	DYS456	DYS458	DYS635	YGATAH4	DYS481	DYS533	DYS549	DYS570	DYS576	DYS643
LM1	15	13	14	13	30	22	10	11	14	16	10	12	22	15	18	21	11	21	10	12	16	15	
LM1.4			14	13		22	10	11	14						18	21		21			16	15	
RM3.2	15	13	14	13	30	22	10	11	14	16	10	12	22	15	18	21	11	21	10	12	16	15	
LM1.3	15	13	14	13	30	22	10	11	14	16	10	12	22	15	18	21	11	21	10	12	16	15	12
RM2.1							10																
RM2.12		13	14	13	30	22	10		14	16		12	22		18	21				12	16	15	

Supplementary Table 5: Y chromosome SNP data for Skeleton 1. Numbers in brackets indicate the number of successful amplifications out of the amplifications attempted. Results for M201, M285 and P287 were further confirmed by sequencing in a second lab.

**Marker**	**Position**	**Alleles detected probes**	**Coverage (probes)**	**Alleles detected: PCR replicates**	**Final Call**	**SNP rs no.**
M170	14,847,792	A	1	A (6/6)	A	rs2032597
M201	15,027,529	T	3	T (6/6)	T	rs2032636
M269	22,739,367	T	2	T (6/6)	T	rs9786153
M89	21,917,313	T	4	T (4/6)	T	rs2032652
M173	15,026,424	A	1	A (4/4)	A	rs2032624
M304	22,749,853	G	1	A (1/4)	?	rs13447352
U152	15,333,149	C	1	C (4/4)	C	rs1236440
M9	21,730,257	C	1	C (4/4)	C	rs3900
M253	15,022,707	C	2	C (1/2)	C	rs9341296
M70	21,893,881	A	1	A (2/2)	A	rs2032672
M198	15030752		/	C (5/6)	C	rs2020857
M285	22741740		/	G (6/6)	G	rs13447378
M96	21778998		/	C (3/4)	C	rs9306841
P128	20837553		/	C (1/2)	?	rs17250121
P131	15472863		/	C (2/2)	C	rs9786043
P132	8679843		/	G (2/2)	G	rs3853054
S116	22157311		/	C (2/2)	C	rs34276300
M242	15018582		/	C (2/2)	C	rs8179021
U106	8796078		/	C (2/2)	C	rs16981293
M231	15469724		/	G (2/2)	G	rs9341278
P214	18747493		/	G (2/2)	G	rs17315680
P287	22072097		/	T (4/6)	T	rs4116820

Supplementary Table 6: Mitochondrial DNA type of ML1, ML2 and Skeleton 1.

**Position**	**Reference**	**Maternal Lineage 1**	**Maternal Lineage 2**	**Grey Friars Skeleton 1**
73	A	G	G	G
146	T	C	C	C
185	G	A	A	A
188	A	G	G	G
263	A	G	G	G
295	C	T	T	T
310	TCCCCC	TCCCCCC	TCCCCCC	TCCCCCC
462	C	T	T	T
489	T	C	C	C
750	A	G	G	G
1438	A	G	G	G
2706	A	G	G	G
3010	G	A	A	A
4216	T	C	C	C
4769	A	G	G	G
7028	C	T	T	T
8860	A	G	G	G
8994	G	G	A	G
10398	A	G	G	G
10685	G	A	A	A
11251	A	G	G	G
11719	G	A	A	A
12397	A	G	G	G
12612	A	G	G	G
13281	T	C	C	C
13708	G	A	A	A
13933	A	G	G	G
14766	C	T	T	T
14798	T	C	C	C
15326	A	G	G	G
15452	C	A	A	A
16069	C	T	T	T
16126	T	C	C	C
16519	T	C	C	C

Supplementary Table 7: Alignment statistics for ancient mitochondrial DNA sequences

**Reference**	**No. of reads processed**	**No. of reads mapped**	**No. of reads mapped after removing duplicates**	**% of duplication**	**No. of unique reads after quality filtering**	**% of unique reads after quality filtering**	**Average depth of coverage**
rCRS, NC_012920.1	115173617	113998224	1207777	99.98	28503	2.3	161

Supplementary Table 8: Mitochondrial SNPs with medical relevance.

**Site**	**Status**	**Action**	**Information**
10398	pathogenic	Resistance to Parkinson's disease	http://omim.org/entry/516002#0002<br>http://www.ncbi.nlm.nih.gov/clinvar/RCV000010359/
12387	pathogenic	Modifier of PINK1 gene - early onset Parkinsons	http://omim.org/entry/516005#0010<br>http://www.ncbi.nlm.nih.gov/clinvar/ RCV000010351.1/

Supplementary Table 9: Hirisplex results for Skeleton 1. Cov = coverage. Numbers in brackets represent number of successful amplifications out of number of amplification attempts. Asterisks indicate likely DNA damage in the sequence.

**Gene**	**Alleles detected: probes**	**Cov.**	**Alleles detected: PCR**	**Total Cov.**	**Final Call**	**SNP**	**Chr.**	**Position**
IRF4	C	9		9	CC	rs12203592	6	396321
KITLG	T	6		6	TT	rs12821256	12	89328335
SLC24A4	A	18		18	AA	rs2402130	14	92801203
TYR	G	11		11	GG	rs1393350	11	89011046
MC1R	G	16		16	GG	rs1805005	16	89985844
MC1R	C	12		12	CC	rs1805006	16	89985918
MC1R	G8/A1 *	9		9	GG	rs2228479	16	89985940
MC1R	G	12		12	GG	rs11547464	16	89986091
MC1R	C6/T3	9		9	CT	rs1805007	16	89986117
MC1R	C	11		11	CC	Y152OCH	16	89986122
MC1R	T	11		11	TT	rs1110400	16	89986130
MC1R	C10/T1*	11		11	CC	rs1805008	16	89986144
MC1R	G	11		11	GG	rs885479	16	89986154
MC1R	G	7		7	GG	rs1805009	16	89986546
SLC45A2	G2/A1*	3	G (6/6)	9	GG	rs16891982	5	33951693
SLC45A2	A2/C1	3	A (7/10)	10	AA	rs28777	5	33958959
EXOC2	C	5	C (4/6)	9	CC	rs4959270	6	457748
TYRP1	A	3	A (5/6)	8	AA	rs683	9	12709305
TYR	A2/C3	5	A (4/6)<br>C (4/6)	13	AC	rs1042602	11	88911696
SLC24A4	T	3	G (5/6)<br>T (5/6)	13	GT	rs12896399	14	92773663
OCA2	C2/T3	5	T (5/6)<br>C (6/6)	16	CT	rs1800407	15	28230318
HERC2	G	2	G (6/6)	8	GG	Rs12913832	15	28365618
MC1R	No insertion	4	No insertion (4/6)	8	no insertion	N29InsA	16	89985753
ASIP/PIGU		0	A (5/6)	5	AA	rs2378249	20	33218090

Supplementary Table 10: Likelihood ratio (LR) and posterior probabilities for H1 under two prior probabilities for five sets of data and two different mtDNA control region databases.

Evidence	LR	Posterior – sceptical prior	Posterior – 50/50 prior
**All**	**6.7 million**	**0.999994**	**0.9999999**
Genetic	79	0.67	0.987
Non-genetic	85,000	0.9995	0.999988
All exc. mtDNA	14,000	0.997	0.99993
All exc. Y	41 million	0.999999	1.0000000
mtDNA only	478	0.92	0.998
For illustrative purposes, below we give likelihood ratios calculated using the European mitochondrial DNA control region database			
mtDNA only	6847	0.994	0.9999
Genetic	1127	0.97	0.999
All	96 million	0.9999996	0.99999999

Supplementary Notes

Supplementary Note 1

Richard III: Historical Information

Supplementary Note 1a

Appearance

There are number of accounts of Richard III's appearance, perhaps the most famous being that given by Shakespeare in his play Richard III, written nearly a hundred years after Richard's death, which describes Richard as being a hunchback with a withered arm and a limp. However, there are only two contemporary accounts of Richard's appearance. These are:

1. 1484, Nicholas von Poppelau's visit to court (itinerant Silesian knight visiting England) Richard was: '*three fingers taller than [Poppelau], but a little slimmer and not as bulky as him, also very much more lean; he had very fine-boned arms and legs, also a great heart*'[1].
2. 1486, John Rous, *Historia Regum Angliae*. Though written after Richard's death, Rous had met Richard. He describes Richard as: 'retained in his mother's womb two years, being born with teeth and hair to the shoulders'; '*He was short of stature, having a short face, **with unequal shoulders, the right higher and the left lower***'[2].

The subsequent descriptions of Richard III being a hunchback could stem from the Rous text.

Supplementary Note 1b

Death and burial

There are a number of accounts, which discuss Richard's death at the Battle of Bosworth and his burial at the Grey Friars friary in Leicester. However, again, it is Rous's *Historia Regum Angliae* which is the most closely contemporary account and which records him as having died in battle and being buried in the choir of the church of the Friars Minor (Franciscans, also known as the Grey Friars) in Leicester.

'However, if I might speak the truth to his honour as a noble soldier, though he was slight in body and weak in strength, to his last breath he held himself nobly in a defending manner, often crying that he was betrayed and saying, 'treason, treason, treason'. And, so tasting what he had more often served to others, he ended his life miserably, and finally he was buried among the Friars Minor (Franciscans) of Leicester in the choir'[2].

Polydore Vergil recorded that *'King Richard, alone, was killed fighting manfully in the thickest press of his enemies'*[3].

Supplementary Note 1c

Population size and number of males killed at the Battle of Bosworth

The population of England and Wales in 1485 at the time of Richard's death was approximately 2.41 million. The figure of 2.41 million is derived by extrapolating from the figures for a population total for England of 1.9 million in 1450 and 2.35 million in 1522 given in Broadberry *et al*. (p.13), and adding 300,000 for Wales [4]. Assuming a typical sex ratio of 1.04 women to men results in an estimated population of 1.18 million men in England and Wales in 1485. The skeleton belongs to a male in the age range 25-34 years. E. A. Wrigley and R.S. Schofield (p.528) give age-specific estimates for 1541[5] . Extrapolating these suggests that men of the age group 24-34 accounted for some 13% of the population at that time. The population structure in 1485 would have been similar to this, yet given the population grew sharply in the period 1522-41 one would expect those aged 25-34 to account for a slightly smaller proportion in 1485[4].This age group would have accounted for, at most, 15% of the population at the time, some 177,206[5]. It is suggested that some 17,000 fought at the battle of Bosworth: 5-7,000 on the side of Henry, and 11-12,000 on that of Richard[6]. Contemporary accounts of the numbers slain at Bosworth vary significantly. Diego de Valera (1486) suggested 'above 10,000 ...on both sides 'Jean Moinet (c.1490) suggested '300...on either side', while Polydore Vergil of Urbino (c. 1502) 'about 1,000'[7]. Today it is generally believed that the casualties were not high, numbering 1,000 in total[8]. It is impossible to be precise about the age structure of the dead. As one might expect, those dying in warfare are generally younger than the population as a whole. Little is known about age-specific mortality rates of those dying in mediaeval conflicts. English and Welsh casualties in the 1st World War – the first for which we have detailed age-specific figures -- numbered 548,747, of which 86.4% were aged less than 35[9]. The age group 25-34 accounted for 37.5% of all war-related deaths. Given the differing age structures of the populations and what is known about medieval warfare, one might reasonable expect those dying at Bosworth to be younger, pro rata, than those of the 1st World War. However, even if we inflate (rather than deflate) the proportion those aged 25-34 dying at Bosworth to 40% of the total number of casualties, this gives an estimated maximum of 400 men of this age slain on the field of Bosworth. In summary, the maximum likely proportion of men nationally in 1485 aged 25-34 who died at Bosworth was 0.225%, or 1:433.

Supplementary Note 2

Genealogical Information

Supplementary Note 2a

Tracing relatives

The DNA identification of any individual relies on comparing their DNA with a known relative. Given the time depth since Richard's death, the way different sections of our DNA are inherited down through the generations and the fact that Richard III left no descendants of his own, this required tracing female-line only relatives and male-line only relatives allowing for mitochondrial and Y chromosomal analyses, respectively. While not carrying Richard III's mitochondrial DNA or Y chromosome (as only Richard could carry his own DNA), modern day relatives could act as comparators for the DNA analysis.

Mitochondrial DNA is passed down by a mother to all of her children: therefore Richard, like all his siblings, would have had his mitochondrial DNA passed down to him through his mother. However, only his sisters would have been able to pass the mitochondrial DNA on. Modern-day individuals related to Richard through the female line should carry an identical or near identical mitochondrial type either through Richard's mother, Cecily Neville, or through earlier maternal relatives.

The Y chromosome carries the sex-determining gene, SRY, and is therefore only passed from male to male down through the generations. It follows that individuals related to Richard through the male line should carry an identical or near identical Y chromosome type through Richard's father or earlier male-line relatives.

Tracing the lineage of those of royal or noble descent is no new field of research and much information has previously been published: important surveys include Richardson[10], Weir[11] and Stuart[12]. In addition to those of royal birth, the ancestry of the peerage, admittedly focusing primarily on the male descent due to the inheritance of titles and positions, has been particularly well covered[13-15]. Alongside this work, extensive work has also been conducted on the genealogies of families more distantly related to either royalty or the peerage. Of particular note is the voluminous work of the Marquis of Ruvigny who attempted to trace all the descendants of Edward III, in his *Plantagenet Roll of the Royal Blood*. His work was published in several volumes over a number of years, and is especially relevant to the reconstruction of the 'Ibsen' line (see later section below)[16]. For the fifteenth and early sixteenth centuries Ruvigny relied heavily on the information recorded in the so-called visitation returns. These documents resulted from visits made between 1530 and 1688 by the officers of arms to various counties in order to check both the social status and to record the pedigrees of those who claimed the right to bear arms. Their written records and notes were then returned to the College of Arms in London[17]. In various instances these have subsequently been transcribed and published, and provide detailed pedigrees of a number of the families relevant to both the 'Ibsen' and 'Duldig' lines[18-20]. These sources were also key to the research of Joseph Foster whose four volume work on the county families of Yorkshire published in 1874/5 was largely based on visitation pedigrees and other documentation, and again provides information relating to both the 'Ibsen' and 'Duldig' lines[21]. The monumental work of Frederick Arthur Crisp whose *Visitation of England and Wales*, published in a total of 35 volumes, including 14 volumes of notes between 1893 and 1921 (Crisp died the following year), also provides an important source of information[22], as does *Burke's Landed Gentry*[23].

Supplementary Note 2b

Close maternal-line relatives of Richard III

Historical accounts record Richard III as having one shoulder higher than the other; sustaining battle injuries and being killed at the Battle of Bosworth; and as being brought back to Leicester and subsequently buried in the choir of the church of the Grey Friars. However, despite there being no record of any of the above being attributed to any of Richard's relatives, could there be any chance that the burial is actually that of a female-line relative of Richard III who also suffered a spinal abnormality, who died at the Battle of Bosworth and who would therefore also show evidence of battle injuries and carry the same mtDNA type?

In order to attempt to answer this question, the inheritance of Richard's mtDNA was traced for seven generations, from his maternal great-great-grandmother down and out through his network of cousins, identifying any males who would have been alive at the time of Bosworth and who might be candidates for the skeleton in the site of the Grey Friars. It is not possible to trace the line further back than this, as the identity of his maternal great-great-great-grandmother is unknown. A summary of this genealogy is given below. In order to simplify the genealogical information, every individual in the overall tree has been assigned a unique number. Additionally, the overall tree has been broken down into a series of component family sub-trees. Females carrying the mtDNA who married and passed this on to children of their own will appear in two family sub-trees, as daughter in the first, then as mother in the second. The descent from sons is not included since, obviously, they cannot pass on the mtDNA. Spouses marrying into the network are included where appropriate, but since these do not carry the mtDNA their identification number is ~~struck through.~~ Males carrying the same mtDNA type as Richard, but NOT at risk of being confused with the skeleton in the Grey Friars friary (as either already dead, known to have survived beyond Bosworth, or alive at the time of Bosworth but clearly too old or too young) have their identification number in **bold**.

This lineage was reconstructed using a wide variety of documentary sources (see covering note on genealogical research above).

Tree 1. The name of Richard III's great-great-grandmother [1] is uncertain. She was the wife of Sir Payne Roët of Guienne (or Paon de Roët) [2]. They had three daughters:

[3] Isabel (Isabelle) became a nun (Canoness of the Convent of St Waudru, Mons) and died childless.

[4] Phillippa (c.1346-c.1387) married the poet Geoffrey Chaucer [~~6~~] when aged about 10. Details of offspring [7-10] are shown below, Tree 2.

[5] Katherine (c.1350-1403), Richard's great-grandmother, who married first Sir Hugh Swynford (c.1366-) [~~11~~], and second, as third wife, John of Gaunt, Duke of Lancaster (-1399) [~~12~~], son of Edward III and father of Henry IV (from his first wife Blanche). Details of offspring [13-19] are shown below, Tree 3.

Tree 2. Phillippa de Roët [4] is recorded as having up to four children, although some suggest only three. It is also possible that some (or all) of these children were actually fathered by John of Gaunt [~~12~~] rather than her husband, Geoffrey [~~6~~][24-26]. All four children are believed to have died childless, the Chaucer family having died out by the 15th century.

[7] Elizabeth (c.1364-), a nun in Barking Abbey.

[**8**] Thomas (c.1367-)

[9] Agnes, a lady in waiting at Henry IV's coronation in 1399.

[**10**] Lewis (c.1381-) (Possibly not their child.)

Tree 3. Katherine de Roët [5] had seven children from her two husbands, three with Hugh Swynford [~~11~~] and four with John of Gaunt [~~12~~] (all born out of wedlock, but subsequently legitimated by charter under Richard II, 1397):

[13] Blanche (1367-1369)

[**14**] Thomas (1368-1432)

[15] Margaret (c.1369-) became a nun and died childless.

[**16**] John Beaufort (1373-1410), 1st Earl of Somerset, Marquis of Dorset and Lord High Admiral of England.

[**17**] Henry Beaufort (c.1374-1447), Bishop of Winchester and Cardinal, Lord Chancellor.

[**18**] Thomas Beaufort (c.1377-1426), 1st Duke of Exeter.

[19] Joan Beaufort (c.1379-1440), who married, first, Sir Robert Ferrers [~~20~~], 5th Baron Botcher of Wem in 1391 and, second, Ralph Neville, 1st Earl of Westmorland [~~21~~]. Details of offspring [22-37] are shown below, Tree 4.

<u>**Tree 4**</u>. Joan Beaufort [19], Richard's grandmother, had sixteen children, two daughters by her first husband, Robert Ferrers [~~20~~], and five daughters and nine sons by her second husband, Ralph Neville [~~21~~], as follows:

[22] Elizabeth Ferrers (1393-1434), who married John de Greystoke [~~38~~], 4th Baron Greystoke (1389-1436), in 1407. Details of offspring [39-50] are shown below, Tree 5.

[23] Margaret Ferrers (1394-1458), who married Ralph Neville [~~51~~] (-1458) in c.1413. The marriage is recorded as resulting in only one son, John Neville [**52**] of Oversley (c.1416-1482), Sheriff for Lincolnshire.

[24] Katherine Neville, (c.1400-c.1484) married four times: to John de Mowbray, 2nd Duke of Norfolk [~~53~~]; Thomas Strangeways [~~54~~], John, Viscount Beaumont; and John Woodville. Details of offspring [55-57] are shown below, Tree 6.

[25] Eleanor Neville, (-1472), who married first, Richard le Despenser [~~58~~], 4th Baron Burghersh, and had no issue, and second, Henry Percy [~~59~~], 2nd Earl of Northumberland. Details of offspring [60-69] are shown below, Tree 7.

[**26**] Richard Neville (1400-1460), 5th Earl of Salisbury.

[**27**] Robert Neville (-1457)

[**28**] William Neville (-1463), 1st Earl of Kent.

[29] Anne Neville, who married Humphrey Stafford [~~70~~], 6th Earl of Stafford and 1st Duke of Buckingham (1402-1460), who died at the Battle of Northampton. Details of offspring [71-80] are shown below, Tree 8.

[**30**] Edward Neville (-1476)

[31] Cecily Neville (1415-1495), mother of Edward IV [86] and Richard III [93], married Richard Plantagenet [~~81~~] (-1460), 3rd Duke of York, Protector of England, died at the Battle of Wakefield. Details of offspring [82-94] are shown below, Tree 9.

[**32**] George Neville (-1469)

[33] Joan Neville, died childless.

[**34**] John Neville, died young.

[**35**] Cuthbert Neville, died young.

[**36**] Thomas Neville, died young.

[**37**] Henry Neville, died young.

__Tree 5__. Elizabeth Ferrers [22] had six sons and six daughters from her marriage with John de Greystoke [~~38~~], 4th Baron Greystoke (c.1389-1436), as follows:

[**39**] Richard, died without issue before his father.

[**40**] Henry, died without issue before his father.

[**41**] William, died without issue before his father.

[**42**] Ralph, (-1487) Baron Greystoke, buried in Kirkham Monastery.

[43] Joan (1408-1456). Married, first, John Darcy [~~95~~] 7th Baron Darcy of Knaith (succeeding his brother Philip), and second, William Stoke [~~96~~]. Details of offspring [97-104] are shown below Tree 10.

[44] Anne (-1477). Married in 1432 to Ralph Bigod [~~106~~] of Settrington, who died at Battle of Towton. Details of offspring [107-112] are shown below, Tree 11.

[**45**] Thomas, died unmarried before 1487 (as brother Ralph succeeded by grand-daughter).

[46] Eleanor, married Ralph Eure [~~115~~]. Details of offspring [116-127] are shown below, Tree 12.

[**47**] John, died unmarried before 1487 (as brother Ralph succeeded by grand-daughter).

[48] Catherine, became a nun and died unmarried.

[49] Matilda, died unmarried.

[50] Elizabeth (1428-). Married Roger Thornton [~~128~~] in 1440. Details of offspring [129-130] are shown below, Tree 13.

Tree 6. Katherine Neville [24] had one son from her first marriage to John de Mowbray [~~53~~] and two daughters from her marriage to Thomas Strangeways [~~54~~], as follows:

[**55**] John de Mowbray (-1461), 3rd Duke of Norfolk.

[56] Joan Strangeways, who married William Willoughby [~~131~~], with whom she had a daughter, Cecily [132], who married Edward Sutton [~~158~~], 2nd Baron Dudley, but had no children born prior to Bosworth.

[57] Catherine Strangeways, who married Henry Grey [~~133~~], 4th Baron of Codnor, yet remained childless.

Tree 7. Eleanor Neville [25] married, first, Richard le Despenser [~~58~~], 4th Baron Burghersh (1396-1414), yet this young marriage was childless. Her second marriage to Henry Percy [~~59~~], 2nd Earl of Northumberland (1393-1455), produced seven sons and three daughters, as follows:

[**60**] John (1418-), died before his father (pre-Bosworth).

[**61**] Henry (1421-1461), 3rd Earl of Northumberland, died at the Battle of Towton. His son, Henry 4th Earl of Northumberland (who would not have shared mtDNA with Richard III), led troops at Bosworth in the Yorkist cause, but failed to engage. After Bosworth he was imprisoned by Henry VII, but subsequently released and allowed to retain his titles and land.

[**62**] Thomas (1433-1460), 1st Baron Egremont, died at Battle of Northampton.

[63] Katherine (1423-1475), married Edmund Grey [~~134~~], 1st Earl of Kent (1416-1490). Details of offspring [135-138] are shown below, Tree 13.

[**64**] George (1424-1474).

[**65**] Ralph (1425-1464), died at Battle of Hedgeley Moor.

[**66**] Richard (1427-1461), died at Battle of Towton.

[**67**] William (1428-1462)

[68] Anne, died unmarried.

[69] Joan, died unmarried.

Tree 8. Anne Neville [29] had six sons, two of whom were twins, and four daughters through her marriage with Humphrey Stafford [~~70~~], 6th Earl of Stafford/1st Duke of Buckingham, as follows:

[**71**] Humphrey (1425-1458), 7th Earl of Stafford. Married Margaret Beaufort (daughter of the 2nd Duke of Somerset) and fathered Henry, 2nd Duke of Buckingham, who was instrumental in persuading Parliament to declare Edward V illegitimate and subsequently offer the throne to Richard III. He later switched his allegiance and rebelled against Richard in favour of Henry Tudor, but the rebellion failed and Buckingham was beheaded for treason in 1483. His widow, Catherine, subsequently married Jasper Tudor.

[**72**] Henry (c.1425-71). Married Margaret Beaufort (daughter of the 1st Duke of Somerset), as Margaret's third husband. She had previously married John de la Pole [~~149~~] (subsequently the 2nd Duke of Suffolk) at a very early age yet the marriage was dissolved before she reached the age of twelve. Her second marriage was to Edmund Tudor, 1st Earl of Richmond, with whom she had Henry Tudor (later Henry VII). Her fourth marriage, following Henry Stafford's death was with Thomas Stanley, 1st Earl of Derby.

[**73**] John (1427-1473), 1st Earl of Wiltshire.

[**74**] Edward, died young.

[75] Margaret (1435-1475). Married Sir Robert Dunham [~~139~~] (1430-) with whom she had a son, Sir John Dunham [**140**] (1450-1524), who was knighted by Henry VII at the Battle of Blackheath in 1497.

[76] Catherine (1437-1476). Married John Talbot [~~141~~], 3rd Earl of Shrewsbury, 3rd Earl of Waterford, 12th Baron Strange of Blackmore (1448-1473). Details of offspring [142-144] are shown below, Tree 15.

[**77**] George (1439-), twin of below, died young.

[**78**] William (1439-), twin of above, died young.

[70] Joan (1442-1484), not known to have married.

[80] Anne (1446-1472), not known to have married.

__Tree 9__. Cecily Neville [31] married Richard Plantagenet [~~81~~], 3rd Duke of York and had thirteen children as follows:

[**82**] Henry (1438-c.1440)

[83] Anne of York (1439-1476), married, first, Henry Holland [~~145~~], 3rd Duke of Exeter, by whom she had a single daughter, Anne [147] who died without issue. Secondly, she married Sir Thomas St Leger [~~146~~], by whom she has a single daughter, Anne St Leger [148], Baroness de Rous, all of whose male children with George Manners were born after the battle of Bosworth. Anne St Leger's daughter, Katherine, is the common maternal ancestor of the two living individuals (Michael Ibsen and Wendy Duldig) from whom DNA samples were taken.

[**84**] Henry (1441-) died in infancy.

[**85**] Edward (1442-1483), Duke of York, later Edward IV.

[**86**] Edmund (1443-1460), Earl of Rutland, died at the Battle of Wakefield.

[87] Elizabeth (1444-1503). Married John de la Pole [~~149~~], 2nd Duke of Suffolk. Details of offspring [150-160] are shown below, Tree 16.

[88] Margaret (1446-1503). Married Charles I [~~161~~], Duke of Burgundy (the Bold) and died childless.

[**89**] William (1447-) died young.

[**90**] John (1448-) died young.

[**91**] George (1449-1478), 1st Duke of Clarence.

[**92**] Thomas (1451-) died young.

[93] Richard (1452-1485), Duke of Gloucester, later Richard III.

[94] Ursula (1455-) died young.

<u>Tree 10</u>. Joan Greystoke [43] is believed to have had five sons and three daughters with her first husband John Darcy [~~95~~], 7th Baron Darcy of Knaith, of Temple Hurst, Yorkshire, as follows. She married, secondly, William Stoke [~~96~~] in 1458, yet had no further children :

[**97**] Richard (1424-1458), married Eleanor Scrope.

[**98**] John (c.1426-1461)

[99] George, died young, unmarried.

[100] Elizabeth, died young, unmarried.

[101] Thomas, died young, unmarried.

[102] Philip, died young, unmarried.

[103] Jane. Married John Beaumont [~~105~~]. Details relating to this marriage are unclear, see Tree 17, are shown below.

[104] Eleanor, died young, unmarried.

<u>Tree 11</u>. Anne Greystoke [44] married Ralph Bigod [~~106~~] of Settrington in 1432 and had six children, two sons and four daughters, as are shown below.

[**107**] John (of Settrington), died at Battle of Towton, 1461.

[**108**] Thomas (c.1435-). Death unknown, but too old to be a skeleton at The Grey Friars friary.

[109] Anne, believed to have died young.

[110] Catherine (c.1439-). Death unknown, believed to have been unmarried.

[111] Matilda (c.1440-). Death unknown, believed to have been unmarried.

[112] Agnes, who married Thomas Stillington [~~113~~], by whom she had a daughter, Catherine [114], whose own sons all died after Bosworth.

However, note that some web-based genealogies record Anne and Ralph as having fifteen children. Yet the Visitation of Yorkshire 1584/85 attributes Ralph Bigod (of Scagglethorphe, next to Settrington) as having nine children through a marriage to Margaret Plumpton (daughter of Sir Robert Plumpton) through whom the line descended. This is presumably either a second marriage or a different Ralph Bigod. None of the children from the second family would have carried the same mtDNA as Richard III.

<u>Tree 12</u>. Eleanor (Ellinor) Greystoke [46], married Ralph Eure [~~115~~] in 1440 and had seven sons and five daughters, as follows. (Although some accounts suggest six of each, the confusion arises because it is not entirely clear if they had two sons called John (*Johanni*) or two daughters called Joan (*Johanna*)). Ralph Eure was killed at the Battle of Towton in 1461:

[116] Joan (c.1438-) died unmarried.

[**117**] William (c.1440-1484). Heir to Ralph. Married Margaret, daughter of Sir Robert Constable of Hainborough.

[**118**] Ralph (c.1442-c.1484). Will proved at York, 19 June, 1484.

[119] Elizabeth (c.1444-1481) died before 1484. Married three times. Details of offspring [164-167] are shown below, Tree 18.

[120] Henry (c.1446-) twin of below, died in childhood.

[**121**] John (c.1446-) twin of below, died in childhood.

[122] Margaret/Margery (c.1448-) unmarried, a nun at Watton.

[123] Robert (c.1450-) was a Knight of the Order of St John of Jerusalem, details of death unknown.

[**124**] John (c.1452-c.93) will proved at York, 11 June, 1493, buried at Hutton Bushell.

[125] Anne (c.1454-) recorded as marrying Thomas Rokeby (who fought at the Battle of Bramham Moor), details of any children unclear but would be too young to be confused with the skeleton 1.

[**126**] Hugh (c.1456-c.1523). Rector of Huggate, and later Brompton in Pickering Lythe. Will proved at York, 16 April, 1523.

[127] Mary (c.1458-) recorded as marrying Hilton (name uncertain) in 1483, details of any children unclear but would be too young to be confused with skeleton 1.

Tree 13. Elizabeth Greystoke [50] married Roger Thornton [~~128~~] in 1440 and had two daughters, as follows:

[129] Elizabeth (c.1450-), married George[~~168~~] Lord Lumley (c.1444-1507). Details of offspring [169-171] are shown below, Tree 19.

[130] Joan, is given as unmarried in some sources, but also could have married Richard Ogle. Any resulting children would be too young to be confused with skeleton 1.

Tree 14. Katherine Percy [63] married Edmund Grey [~~134~~], 1st Earl of Kent and had two sons and two daughters as follows:

[**135**] Anthony, married Eleanor, sister of Elizabeth Woodville, wife of Edward IV, and died childless in 1480.

[**136**] George (1454-1505), 2nd Earl of Kent.

[137] Elizabeth (-1472) married Sir Robert de Greystock [~~172~~] (c.1443-1483) and died without having any sons.

[138] Anne, married John Grey [~~173~~], 8th Baron Grey of Wilton (c.1443-1499). Details of offspring [174-183] are shown below, Tree 20.

Tree 15. Catherine Stafford [76] married John Talbot [~~141~~], 3rd Earl of Shrewsbury, 3rd Earl of Waterford, 12th Baron Strange of Blackmore (1448-1473) and had two sons and a daughter:

[**142**] George (1468-1528) 4th Earl of Shrewsbury, 4th Earl of Waterford, 10th Baron Talbot, 9th Baron Furnivall (1468-1528). Fought with Henry at Bosworth, as did his uncle and guardian, Sir Gilbert Talbot.

[**143**] Thomas (1470-) believed to have died young, but too young to be skeleton 1.

[144] Anne (1472-) married Thomas Butler. Any children would have been born post Bosworth.

<u>Tree 16</u>. Elizabeth of York [87] married John de la Pole [~~149~~], 2nd Duke of Suffolk and had seven sons and four daughters:

[**150**] John de la Pole (c.1462-1487), 1st Earl of Lincoln. Married Lady Margaret FitzAlan who together has a son (Edward) who died young. He was the *de facto* heir to Richard III (his maternal uncle) following Bosworth, yet initially sided with Henry VII. Subsequently he led a short-lived Yorkist rebellion and was defeated and killed at the Battle of Stoke.

[**151**] Geoffrey (1464-) died young.

[**152**] Edward (1466-1485) Archdeacon of Richmond.

[153] Elizabeth (c.1468-1489) married Henry Lovel, 8th Baron Morley, yet had no children.

[**154**] Edmund (1471-1513) 3rd Duke of Suffolk, beheaded by Henry VIII as a Yorkist pretender.

[155] Dorothy (1472-) died young.

[**156**] Humphrey (1474-1513), a cleric.

[157] Anne (1476-1495) died unmarried, a nun.

[158] Catherine (c.1477-1513) married William Stourton, 5th Baron Stourton, yet had no children.

[**159**] William (1478-1539) of Wingfield Castle, yet often kept in the Tower of London. Married Katherine Stourton but had no children.

[**160**] Richard (1480-1525) lived in exile as a Yorkist pretender following death of his brothers, allying himself with Louis XII of France. Two planned invasions, however, never took place and he died fighting alongside Francis I at the Battle of Pavia. Was known as the 'White Rose'.

<u>Tree 17</u>. Jane Darcy [103] married John Beaumont [~~105~~]. Details relating to this marriage are unclear, yet Foster *County Families* [21] gives a John Beaumont of Newsome as marrying Jane, who could be Jane Darcy. This marriage resulted in two sons, Adam and Henry, both of whom were living during the reign of Henry VII. Some sources suggest that Jane's marriage was dissolved.

<u>Tree 18</u>. Elizabeth Eure [119] married three times, first to Sir William Bulmer [~~162~~], second to Sir James Strangeways [~~163~~], third to John Ellerker [~~164~~]. The first marriage resulted in a daughter, the second marriage a daughter and two sons, whilst the third marriage was childless:

[164] Anne, no details are known, but if she had had a son he would certainly have been too young to have fought at Bosworth.

[165] Felicia (1467-) married William Aske (1465-1512) in 1482. Their daughter, Alice was born after Bosworth.

[**166**] Ralph, born after 1469 and therefore too young to be skeleton 1.

[**167**] Edward, born after 1469 and therefore too young to be skeleton 1.

Tree 19. Elizabeth Thornton [129], married George[~~168~~] Lord Lumley and had three sons:

[**169**] Thomas (1460-1487, buried in Lumley, Durham) who married Elizabeth Plantagenet, illegitimate daughter of Edward IV and Elizabeth Waite. Their son, Richard, became 3rd Baron Lumley.

[**170**] Roger (1475-1530)

[**171**] Ralph, whose death is unknown, but would have been aged less than 10 at the time of Bosworth.

Tree 20. Anne Grey [138], married John Grey [~~173~~], 8th Baron Grey of Wilton (c.1443-1499). Richardson, *Royal Ancestry* [10] records them as having seven sons [174-179] and three daughters [180-183]. However, other sources record John Grey marrying secondly, Elizabeth Vaughan, daughter of Sir Thomas Vaughan and widow of Sir Thomas Cokesey, with the first marriage resulting in only one child, Edmund:

[**174**] Reginald, died before his father in 1499.

[**175**] Edmund (c.1469-1511) became 9th Baron.

[**176**] Richard, details uncertain but he would have been aged 14 or younger at the time of Bosworth.

[**177**] Peter, details uncertain but he would have been aged 14 or younger at the time of Bosworth.

[**178**] Edward, details uncertain but he would have been aged 14 or younger at the time of Bosworth.

[**179**] George, details uncertain but he would have been aged 14 or younger at the time of Bosworth.

[**180**] Thomas, details uncertain but he would have been aged 14 or younger at the time of Bosworth.

[181] Jane (1473-) married Sir Watkin Vaughan. Any children would have been born after Bosworth.

[182] Katherine (1475-) married Sir Thomas Rotherham. Any children would have been born after Bosworth.

[183] Tacy (c.1484-) married John Gyse. Any children would have been born after Bosworth.

What does the information from this web of relatives descended from Sir Payne Roët and his wife tell us? It traces seven generations of descendants and has identified 144 individuals who would have shared the same mtDNA as Richard III, of whom 82 were male, excluding Richard himself. Of these males, 81

could not be skeleton 1 as they either are known to have died either pre or post Bosworth, or were clearly either too old or too young at the time of Bosworth (1485). This leaves just one candidate male: Robert Eure, born around 1450 (Richard was born in 1452), whose death is unknown. However, there is no record of the family having fought at Bosworth, and being a Knight of the Order of St John of Jerusalem (Knights Hospitaller), he is likely to have spent time in the Mediterranean, especially Rhodes, and could even have died there. In addition, there are 4 females who *could* in theory have passed on the mtDNA to a future generation but for whom no clear details are known. However, importantly, no record of any marriage for any of these women is recorded, so it is reasonable to assume that they did not have children.

Supplementary Note 2c

Descendants of Richard III: none survive to the present day

Richard III left no descendants. Historical records indicate that Richard had one legitimate son (Edward, died 1484) through his marriage to Anne Neville, and two illegitimate children, John of Gloucester (thought to have been executed, on the orders of Henry VII in 1499) and Katherine Plantagenet who appears to have died shortly after her marriage to William Herbert, Earl of Huntingdon[27]. As such, no descendants of Richard III survive to the present day.

Supplementary Note 2d

Modern day maternal-line relatives

The University of Leicester acknowledges the important contributions of David Annal and Dr Morris Bierbrier, both of whom are independent genealogical experts. The former was previously Principal Family History Specialist at the Family Records Centre, The National Archives. The latter is a Fellow of the Society of Genealogists, specialising in royal lineage. Additional archival research was undertaken by Bob Matthews in New Zealand.

The majority of the family tree of Anne of York down the maternal line to Joy Ibsen and her children was published as separate parts of Ruvigney's *Plantagenet Roll* in 1907[16] and also featured in various tables in Foster's earlier work on Yorkshire county families[21]. Indeed, in a later separate volume, Ruvigney published a single tree showing the Ibsen line from Anne of York all the way to Barbara Gough (died 1826) including her marriage to Isaac Spooner (Table showing the descent of George Brooks Percy Lillington' and 'Table showing the descent of Major Hugh De Crespigny Huntsman')[28]. Subsequently, and most significantly for this project, John Ashdown-Hill traced the lineage further down to Joy Ibsen, and her children Michael, Jeff and Leslie[29]. Joy Ibsen was the 16th generation great-niece, down the female line, of Richard III. By finding Joy, Ashdown-Hill had found someone who could act as a potential comparator for mtDNA analysis (see more on the DNA below) should any putative remains of Richard III be found. Joy Ibsen died in 2008, but her son, Michael Ibsen, very kindly agreed to take part in the project.

Supplementary Note 2e

Maternal line genealogical work for this project

As is clear from the previous section, the Ibsen line and the identity of Michael Ibsen were known prior to the excavation due to the work of John Ashdown-Hill. However, since the provenance of the exhaustive work of Ruvigny on the Plantagenet lineage is often vague and the subsequent work of Ashdown-Hill extending the lineage of Anne of York down to Joy Ibsen provides no supporting documentary evidence, it was important to validate the Ibsen line independently. This was seen as central to the planned work since should there be a mismatch between the skeleton's DNA and that of Michael Ibsen, this could be due to the fact that the published Ibsen lineage was in error.

Equally, and for similar reasons, from the start of the project, we felt that it was important to try and establish a second lineage in addition to the Ibsen line, so that any subsequent results could be triangulated. As a result, alongside the research to verify and document Michael Ibsen's lineage, Schürer (assisted by Annal, Bierbrier and Matthews) traced and documented a second female-line relative of Richard III, Wendy Duldig. The results of this research are shown in the following sections with documentary and archival information supporting the research shown beneath each individual entry. Please see Supplementary Figure 1 for a detailed genealogy.

Supplementary Note 2f

The Ibsen Lineage

See Supplementary Figure 1

1. Anne St. Leger
Anne was born in 1476 in Ulcombe, Kent. Her parents were Thomas ST. LEGER and Anne of York, the Duchess of Exeter and sister of Richard III. She married Sir George MANNERS around 1495 and was buried in St George's Chapel, Windsor in 1526. Her will was written on 20 April 1526 and proved on 24 February 1526/27.

W. B. Bannerman, (ed) *The Visitations of Kent Taken In The Years 1530-1, and 1574 [Part I (A-H), Part II (I-Z)], and the Visitation Of Kent 1592*, (Harleian Society, 1923-4), vol. 75. http://www.stgeorges-windsor.org/archives/blog/?tag=rutland-chantry-chapel. Will and probate of Anne Lady Roos. Proved, London (PCC) 24 February 1526 (The National Archives (TNA) reference PROB11/22 f.127).

2. Catherine MANNERS
Catherine was born around 1510 to George MANNERS 12th Baron de ROS (or ROOS) and Anne (née ST. LEGER). She is mentioned in her mother's will as 'Kateryn my daughter'. Around 1520, Catherine married Robert CONSTABLE and following her death was apparently buried at 'Babthorpe Chapel' in the parish of Hemingbrough, Yorkshire.

TNA PROB11/22 f.127, lines 6 and 23.

3. Barbara CONSTABLE
Barbara was born around 1530 and was the daughter of Sir Robert CONSTABLE and Catherine (née MANNERS). She married William BABTHORPE around 1550 and died before 1564 since William married

his second wife, Frances Dawney that year. William died later in 1581 and it is noted that he wished to buried alongside his wife (Barabara) in Hemingborough. A grant of letters of administration was made at the Prerogative Court of York on 8 June 1581.

J. T. Cliffe, 'Babthorpe family (*per. c.*1501–1635)', *Oxford Dictionary of National Biography*, Oxford University Press, 2004; online edn, Jan 2008 [http://www.oxforddnb.com/view/article/71868] doi:10.1093/ref:odnb/75309. T. Burton, *The History and Antiquities of the Parish of Hemingbrough in the County of York*, (1888) p.314, states that 'His eldest son, another Sir William, died in 1581, leaving a will, which does not now exist, in which he desired to be buried in his chapel at Hemingbrough, near the body of Barbara his wife.'. Index of Wills in the York Registry, 1568-1585. *Yorkshire Archaeological Society*, **19** (1895) p.187.

4. Margaret BABTHORPE
Margaret was born around 1550. Her parents were Sir William BABTHORPE and Barbara (née CONSTABLE). She married Sir Henry CHOLMLEY or CHOLMONDLEY around 1575 and was buried at St John's, York on 15 April 1628.

Cliffe, 'Babthorpe family'. http://www.historyofparliamentonline.org/volume/1558-1603/member/cholmley-henry-1556-1616

5. Barbara CHOLMLEY or CHOLMONDLEY
Barbara was born around 1575. She married Thomas Belasyse, 1st Viscount Fauconberg of Henknolwe in 1601. Barbara died in 1618. Her husband died around 1653, leaving a will which was written on 9 September 1652 and proved at the PCC on 12 July 1653.

C. M. Newman, 'Bellasis family (*per. c.*1500–1653)', *Oxford Dictionary of National Biography*, Oxford University Press, 2004; online edn, Jan 2008 [http://www.oxforddnb.com/view/article/71863]. doi:10.1093/ref:odnb/75367. Will and probate of Thomas Lord Fauconberg. Proved, London (PCC) 12 July 1653 (TNA reference PROB11/226 ff.102-103).

6. Barbara BELASYSE or BELLASIS
Barbara was baptised on 12 October 1609, the daughter of Thomas BELASYSE, 1st Viscount Fauconberg of Henknolwe and his wife Barbara (née CHOLMLEY). She married Henry SLINGSBY on 7 July 1631 at St Mary Abbot's, Kensington. According to Henry SLINGSBY's diary, Barbara died on 31 December 1641. Neither Henry nor Barbara left a will.

England, Births and Christenings, 1538-1975, index, *FamilySearch* (https://familysearch.org/pal:/MM9.1.1/NPB1-K57), Barbarie Belassis, 12 Oct 1609; citing COXWOLD,YORK,ENGLAND, reference ; FHL microfilm 844560. England, Marriages, 1538–1973, index, *FamilySearch* (https://familysearch.org/pal:/MM9.1.1/NLQ4-CZ2), Henry Slingby and Barbara Belasys, ; citing Saint Mary Abbots,Kensington,London,England, reference ; FHL microfilm 845231. D. Scott, 'Slingsby, Sir Henry, first baronet (1602–1658)', *Oxford Dictionary of National Biography*, Oxford University Press, 2004 [http://www.oxforddnb.com/view/article/25727] doi:10.1093/ref:odnb/25727. Rev D. Parsons, (ed.) *The diary of Sir Henry Slingsby of Scriven,* (1836) pp.73-4.

7. Barbara SLINGSBY
Barbara was born on 16 May 1633, the daughter of Sir Henry SLINGSBY and his wife Barbara (née BELASYSE). She married Sir John TALBOT on 13 July 1660 at St Peter, Paul's Wharf in the City of London.

Sir John died sometime around 1714. His will was written on 1 August 1712 and proved on 30 May 1714. Barbara would appear to have died before her husband as she is not named in his will.

Parsons, *Sir Henry Slingsby*, p.3. England, Marriages, 1538–1973 , index, *FamilySearch* (https://familysearch.org/pal:/MM9.1.1/NKDG-SFM), John Talbott and Barbera Slingsby, ; citing Saint Peter Pauls Wharf,London,London,England, reference ; FHL microfilm 547508. Will and probate of Sir John TALBOT, proved, London (PCC) 30 May 1714 (TNA reference PROB11/540 ff.125-128)

8. Barbara TALBOT

Barbara was born around 1665. She was the daughter of Sir John TALBOT and his wife Barbara (née SLINGSBY) and is mentioned in her father's will as his 'second daughter Barbara'. Later on the will mentions 'my Grandson Talbot Lord Viscount Longueville (eldest son of my Daughter Barbara Viscountess Longueville)'. She married Henry YELVERTON (15th Baron Grey de Ruthyn, 1st Viscount Longueville) on 11 July 1689 at St Martin in the Fields, Westminster. His widow, Barbara (née TALBOT) lived to the age of 98, dying on 31 January 1763 at Brandon, Warwickshire. She left a will which was written on 13 July 1759 and proved at the PCC on 5 February 1763.

Will and probate of Sir John TALBOT. Proved, London (PCC) 30 May 1714 (TNA reference PROB11/540 f.1256r lines 34-35). England, Marriages, 1538–1973 , index, *FamilySearch* (https://familysearch.org/pal:/MM9.1.1/NJYN-25C : accessed 30 Jan 2013), Henry Grey and Barbara Talbot, ; citing Saint Martin In The Fields,Westminster,London,England, reference ; FHL microfilm 561155. *Gentleman's Magazine*, **33**, (February 1763), p.97. Will and probate of Barbara Viscountess LONGUEVILLE. Proved, London (PCC) 5 February 1763 (TNA reference PROB11/884 ff.161-2).

9. Barbara YELVERTON

Barbara was born around 1692. Her parents were Henry YELVERTON and his wife Barbara (née TALBOT). She is mentioned in her father's will as 'my daughter Barbara'. The will also indicates that she was his eldest daughter. Barbara married Reynolds CALTHORPE in 1715, by a special licence which was granted at the Faculty Office on 11 June 1715. Reynolds CALTHORPE died on 12 April 1719 and was buried at Elvetham, Hampshire. He left a will which was proved at the PCC on 3 June 1720. His widow, survived him, being buried on 2 June 1724 at Elvetham, dying intestate.

Will and probate of Henry Viscount LONGUEVILLE. Proved, London (PCC) 1 June 1704 (TNA reference PROB11/477 ff.9-10), see lines 8-9 and 37. J. L. Chester, (ed.), *The Marriage, Baptismal, and Burial Registers of the Collegiate Church or Abbey of St. Peter, Westminster*, (London, England: Harleian Society, 1876), p.19, fn 15. Will and probate of Reynolds CALTHORPE. Proved, London (PCC) 3 June 1720 (TNA reference PROB11/574 ff.252-253).

10. Barbara CALTHORP

Barbara was born around 1716. Her parents were Reynolds CALTHORP(E) and his wife Barbara (née YELVERTON). She is mentioned as a beneficiary in the will of her grandmother, Barbara Talbot as 'my Grand-daughter the Lady Gough'. And also that of her father, in which she is referred to as 'my daughter Barbara'. On 2 July 1741 she married Henry GOUGH at the parish church of St Martin in the Fields, Westminster. Sir Henry died in 1774 and was buried at Edgbaston, Warwickshire on 15 June 1774. His will was proved at the PCC on 2 July 1774. Dame Barbara survived Henry, being buried at Edgbaston on 22 April 1782.

TNA PROB11/884 f.161v lines 31-32. TNA PROB11/574 f.253r line 16. *Gentleman's Magazine*, **11**, July 1741 p.387. D. Cannadine, 'Calthorpe family (*per.* 1717–1910)', *Oxford Dictionary of National Biography*, Oxford University Press, Sept 2013 [http://www.oxforddnb.com/view/article/106104] doi:10.1093/ref:odnb/106133. National Burial Index, 3rd Ed. Will and probate of Sir Henry GOUGH. Proved, London (PCC) 2 July 1774 (TNA reference PROB11/999 ff.245-247).

11. Barbara GOUGH
Barbara was baptised at St. George's, Hanover Square, Westminster on 12 April, 1746 (born 21 March). Her parents were Sir Henry GOUGH and his wife Barbara (née CALTHORP). She is mentioned in the will of her mother as 'my Daughter Barbara Spooner'. She married Isaac SPOONER in Edgbaston on 9 January 1770, banns having also been called at Elmdon parish church (Isaac's home parish) on the three preceding Sundays. Isaac died in 1816 and was buried at Elmdon on 14 June 1816. He left a will which was proved at the PCC on 23 October 1816. Barbara survived Isaac, eventually dying in 1826. She was buried at Elmdon on 4 April 1826, leaving a will which was written on 17 November and 1820 and proved at the PCC on 20 June 1826.

http://search.findmypast.co.uk/record?id=gbprs%2fwsmtn%2f005620058%2f00353&parentid=gbprs%2fb%2f491686374%2f1&highlights=%22%22. Will and probate of Barbara GOUGH. Proved, London (PCC) 3 May 1782 (TNA reference PROB 11/1090 ff.268-269), f.268r lines 14-15. England, Marriages, 1538–1973 ," index, *FamilySearch* (https://familysearch.org/pal:/MM9.1.1/V5V5-H7Q : accessed 30 Jan 2013), Isaac Spooner and Barbara Gough, ; citing Edgbaston,Warwick,England, reference ; FHL microfilm 1426213 IT 1-2, 502272, 502273, 502274. Elmdon parish registers (Warwickshire CRO reference PG2984 DR(B)14/2). National Burial Index, 3rd Ed. Will and probate of Isaac SPOONER. Proved, London (PCC) 23 October 1816 (TNA reference PROB11/1585 ff.105-114). Will and probate of Barbara SPOONER. Proved, London (PCC) 20 June 1826 (TNA reference PROB11/1585 ff.376-378).

12. Ann SPOONER
Ann was baptised on 16 February 1780 in the parish church of Aston juxta Birmingham, the daughter of Isaac SPOONER and his wife Barbara (née GOUGH). She is mentioned in the will of her mother as 'my daughter Ann the wife of the Reverend Edward Neale'. She married Edward NEALE who changed his name from VANSITTART by Royal Licence on 14 November 1803. He was the Rector of Taplow, Bucks., from 1803 until his death in 1850. In the 1841 census the family were living at the Vicarage in Taplow. Edward died in 1850. His will was written on 2 September 1848 and proved at the PCC on 27 February 1850. Following Edward's death, Ann moved to Hastings. She is listed there in the 1851, 1861 and 1871 censuses. Ann died in Hastings in 1873 and her will was proved in London on 27 December 1873.

England, Births and Christenings, 1538-1975, index, *FamilySearch* (https://familysearch.org/pal:/MM9.1.1/JM1X-H83 : accessed 30 Jan 2013), Ann Spooner, 16 Feb 1780; citing Edgbaston,Warwick,England,, reference ; FHL microfilm 919792, 919793. TNA PROB11/1585 f.377r lines 15-16. 1841 census (TNA reference HO107/48/11 f.5 p.3). Will and probate of Edward NEALE. Proved, London (PCC) 27 February 1850 (TNA reference PROB11/2107 ff.344-346). 1851 census (TNA reference HO107/1635 f.426 p.63 & f.427 p.64). 1861 census (TNA reference RG9/560 f.64 p.51). 1871 census (TNA reference RG10/1031 f.54 p.36). Will and probate of Ann NEALE. Proved, London 27 December 1873 (Principal Registry of the Family Division (PRFD)).

13. Charlotte Vansittart NEALE
Charlotte was baptised in Taplow, Buckinghamshire on 21 April 1817, the daughter of Edward NEALE and Ann (née SPOONER). She is mentioned in her father's will as 'my daughter Charlotte Vansittart Frere' as

well as that of her mother. In 1841 she married Charles FRERE. The family were living in Croydon (Stroud Green, Shirley) in 1851 and 1861 but by 1871, they had moved to Paddington (London). Charlotte died in January 1881 in Bournemouth leaving a will which was proved at London on 25 May 1881. Charlotte's husband, Charles FRERE, died in 1884. Her brother, Edward, was a leading figure in the Christian Socialist Co-operative movement.

England, Births and Christenings, 1538-1975, index, *FamilySearch* (https://familysearch.org/pal:/MM9.1.1/JMBD-J43 : accessed 30 Jan 2013), Charlotte Vansittart Neale, 21 Apr 1817; citing TAPLOW,BUCKINGHAM,ENGLAND, reference ; FHL microfilm 919250. TNA PROB11/2107 f.345v lines 2-3. Will and probate of Ann NEALE. Proved, London 27 December 1873 (PRFD). General Register Office (GRO) Index to Marriages registered in Q4 1841, Registration District: Eton, Volume: 6, Page: 655. 1851 census (TNA reference HO107/1601 f.529 p.8). 1861 census (TNA reference RG9/450 f.195 p.3).1871 census (TNA reference RG10/22 f.36 p.25). Will and probate of Charlotte Vansittart FRERE. Proved, London 25 May 1881 (PRFD). GRO Index to Deaths registered in Q1 1881, Registration District: Christchurch, Volume: 2b, Page: 430, where she is registered as aged 63 at death which tallies with her birthdate. Matthew Lee, 'Neale, Edward Vansittart (1810–1892)', *Oxford Dictionary of National Biography*, Oxford University Press, 2004; online edn, May 2005 [http://www.oxforddnb.com/view/article/19820] doi:10.1093/ref:odnb/19820.

14. Charlotte Vansittart FRERE
Charlotte was born in Westminster in 1846, the daughter of Charles FRERE and Charlotte Vansittart (née NEALE). She is mentioned in the will of her father: 'my Children … Charlotte Vansittart Frere…'. The family were living in Croydon (Stroud Green, Shirley) in 1851 and 1861, moving to Paddington, London, by 1871. Charlotte married Allen Folliot STOKES in 1882 and died in 1916.

GRO Index to Births registered in Q2 1846. Registration District: Westminster, Volume: I, page 447. Will and probate of Charles FRERE. Proved, London 21 April 1884(PRFD). 1851 census (TNA reference HO107/1601 f.529 p.8). 1861 census (TNA reference RG9/450 f.195 p.3). 1871 census (TNA reference RG10/22 f.36 p.25). GRO Index to Marriages registered in Q4 1882, p.104. Registration District: Wokingham, Volume: 2c, Page 818. GRO Index to Deaths registered in Q1 1916, p.176. Age: 69, Registration District: Newton A [Newton Abbot], Volume: 5b ,Page: 205.

15. Muriel Charlotte Folliot STOKES
Muriel was born in 1884, the daughter of Alan (Allen) Folliot Gardiner STOKES and Charlotte Vansittart (née FRERE). At the time of the 1891 census Muriel was living in St Ives, Cornwall with her parents who were listed in the returns as 'visitors'. In 1901, Muriel was in Penzance with her mother. Muriel's marriage (to Orlando Moray BROWN) took place in 1919. She died in Canada in 1961.

GRO Index to Births registered in Q2 1884 p.523. District: Kingston,Volume: 2a,Page: 307. 1891 census (TNA reference: RG12/1854 f.146 p.8). 1901 census (TNA reference: RG13/2254 f.124 p.2). GRO Index to Marriages registered in Q2 1919 p.251. District: Newton A, Volume: 5b, Page: 307.

16. Joyce Muriel BROWN – also known as Joy IBSEN
Joyce's birth was registered in the Paddington (London) registration district in 1926. She is mentioned in the will of her grandfather and that of her mother. She moved to Canada with her mother in 1948, travelling on-board the SS *Mauretania* in August. Joy BROWN married Norm IBSEN and the couple had three children, Michael, Jeff and Leslie. Joy Ibsen died in 2008.

Ashdown-Hill ('Alive and Well', fn. 38) notes that she was registered as Murial J. Brown. This was indeed the case (GRO Index to Births registered in Q2 1926 p.143. District: Paddington, Volume: 1a, Page: 106), but actually the birth was rather oddly later re-registered (GRO Index to Births registered in Q2 1926 p.144. District: Paddington, Volume: 1a, Page: 106.A) switching the order of the names. Will and probate of Allen Folliot Gardiner STOKES. Proved, London 5 January 1940 (PRFD). Allen Folliot Gardiner STOKES names his grandchildren, Kenneth Murray Brown, Patrick Murray Brown and Joyce Murray Brown, as his sole beneficiaries. Although the middle names are all wrong, it is clear that these are the three children of his daughter Muriel. Moray (not Murray) was the middle name of Allen's son-in-law, Orland BROWN. Will and probate of Muriel Charlotte Folliot BROWN. Proved, London 28 December 1961 (PRFD). This names her daughter Joy Ibsen as her sole executrix - 'my Son & daughter Patrick Brown and Joy Brown who is now Mrs Ibsen'. Passenger Lists (Passengers Leaving the UK) TNA reference BT27/1630.

Supplementary Note 2g

The Duldig Lineage

See Supplementary Figure 1

1. Anne St. Leger (as above)

2. Catherine MANNERS (as above)

3. Everhilda CONSTABLE
Everhilda, the sister of Barbara (see above), was born in Everingham, Yorkshire around 1535 the daughter of Sir Robert CONSTABLE (d. 1558) and Catherine (née MANNERS). She was married at Everingham in c.1561 to Thomas CRATHORNE of Crathorne, Yorkshire.

Norcliffe, *Visitation of Yorkshire*, p.66, where it is noted that 'these four daughters [including Everhilda – given as Everell] are wrongly made daughters of Sir Marmaduke Constable, their grandfather, in Foster, *Visitation of Yorkshire*, p. 198. J. Graves, *The History of Cleveland in the North Riding of Yorkshire*, (1808), p.107.

4. Katherine CRATHORNE
Katherine's date of birth is unknown but was probably c1555. Her parents were Thomas CRATHORNE and Everhilda (née CONSTABLE). She married Ralph Creyke of Marton, Yorkshire who was buried on 27 July 1623. She was buried on May 6, 1606.

Foster, *Visitation of Yorkshire*, p. 163 and B. Burke, *A Genealogical and Heraldic History of the Commoners of Great Britain*, (1838), vol. 4, p.25.

5. Everhilda CREYKE
Daughter of Ralph CREYKE and Katherine (née CRATHORNE) married Christopher MALTBY of Maltby, Yorkshire in 1600.

Foster, *Visitation of Yorkshire*, p. 163 and Burke, *Genealogical and Heraldic History of the Commoners*, vol. 4, p.25.

6. Everhilda MALTBY

Everhilda was born in 1605, the daughter of Christopher MALTBY and his wife Everhilda (née CREYKE). She was married in 1626 to Sir George WENTWORTH of Woolley, Yorkshire, MP, and died in c.1670.

J. W. Clay, (ed.) *Dugdale's Visitation of Yorkshire with additions*, (1907) vol. 2, pp.322-2.

7. Frances WENTWORTH

Frances was baptised at Woolley on 1 December, 1631. She was married at Woolley on 12 January 1658 to Thomas GRANTHAM of Meux Abbey, Yorkshire. She died on the12 March, 1693.

Woolley parish register.A. R. Maddison, *Lincolnshire Pedigree,* (1903) vol. 2, p.423 and Clay, *Dugdale's Visitation* , vol. 2, pp.322-2.

8. Dorothy GRANTHAM

Dorothy was baptised at Woolley on 28 April 1659 the daughter of Thomas GRANTHAM and Frances (née WENTWORTH). She married James HOLT of Castleton, near Rochdale, Lancashire, MP, on 24 February 1679 and died in 1717.

Woolley parish register. A.R.Maddison, *Lincolnshire Pedigree,* vol. 2, p.423. B. Hennig, (ed.) *History of Parliament. The Commons 1660-1690*, (1983), vol. 2, pp. 571-2. Deed of 1698, Lincoln Record Office, Tur/11/1/6/4.

9. Frances HOLT

Frances was baptised at Rochdale on 28 March 1681 the daughter of James HOLT and Dorothy (née GRANTHAM). She was married in c. 1701 to James WINSTANLEY (who was MP for Braunstone, Leicestershire between 1701-19) and died 1771.

Maddison, *Lincolnshire Pedigree,* vol. 2, p.423. Hennig, *The Commons 1690-1715*, (2002), vol. 5, p.903. Burke, *A Genealogical and Heraldic History of the Commoners*, vol. 1, p.364. Rochdale parish register.

10. Frances WINSTANLEY

Frances was born in c.1702/3 the daughter of James WINSTANLEY and Frances (née HOLT). She was married to the brewer Sir Benjamin TRUMAN of Pope's Manor, Hertingfordbury, Hertfordshire. She died on 10 June 1766.

Burke, *A Genealogical and Heraldic History of the Commoners,* vol. 1, p. 364 where her name is wrongly given as Anne –Letitia; correctly on her tombstone - J. Parker, (ed.) *Monumental Inscriptions of St Mary's Hertingfordbury*, (1989), No. 635. P. Mathias, 'Truman, Sir Benjamin (1699/1700–1780)', *Oxford Dictionary of National Biography*, Oxford University Press, 2004; online edn, Jan 2011 [http://www.oxforddnb.com/view/article/50468] doi:10.1093/ref:odnb/50468. Will of Benjamin Truman TNA reference PROB 11/1064.

11. Frances TRUMAN

Frances was born in 1726 the daughter of Benjamin TRUMAN and Frances (née WINSTANLEY). She was married on 27 March 1747 to Henry READ of Crowood, Ramsbury, Wiltshire. She is mentioned in the will of her father, Benjamin Truman as 'my only child, Frances Read, wife of Henry Read'. She died in 1801. She is also mentioned in the wills of her husband and daughter.

Faculty Office Marriage Licence 27 March 1747. TNA PROB 11/1064 f.221r lines 18-19.
TNA PROB 11/1143/394 will of Henry Read. TNA PROB 11/1358/115 will of Frances Read.

12. Frances READ

Frances Read was born in 1750 the daughter of Henry READ and Frances (née TRUMAN). She was married at Ramsbury on 31 October, 1768 to William VILLEBOIS of Feltham Place, Middlesex, her former dance teacher. She is mentioned in the will of her grandfather, Benjamin Truman as my 'Grand Daughter Frances Villebois and W. Villebois her husband'. She died in 1820. Her portrait was painted by Thomas Gainsborough (following her marriage), commissioned by her grandfather, Benjamin Truman. The painting was sold at auction in July, 2011. Gainsborough also painted portraits of her grandfather, her sister, Henrietta, and her two sons, John and Henry (jointly).

British Library, 17th and 18th newspapers online, *The Morning Post,* 13 May 1768. TNA PROB 11/1064 f.227 lines 12-13. Ramsbury parish register. http://www.christies.com/lotfinder/paintings/thomas-gainsborough-ra-portrait-of-mrs-william-5460654-details.aspx. http://www.tate.org.uk/art/artworks/gainsborough-sir-benjamin-truman-t02261/text-catalogue-entry. http://emuseum.huntington.org/view/objects/asitem/People$00403287/0?t:state:flow=2d7f3a49-af78-4c4b-9b76-fd2fe010a5bf

13. Harriet VILLEBOIS

Harriet was born in 1774 and baptised on 15 December at Feltham, Middlesex, the daughter of William VILLEBOIS and Frances (née READ). She was married at Lymington on 31 January, 1805 to Charles Cornelius PLUNKET. She died in Southampton 6 Oct 1821.

http://search.findmypast.co.uk/record?id=gbprs%2fb%2f907039899%2f1&highlights=%22%22. British Library 19th century newspapers *The Morning Post* ,5 Feb. 1805. http://search.findmypast.co.uk/record?id=gbprs%2fm%2f810066678%2f4&highlights=%22%22. http://www.clanbarker.com/placesearch.php?psearch=Rosehill+House%2C+Southampton%2C+Hampshire.

14. Harriet PLUNKET

Harriet Maria Elizabeth was born in Florian, Malta on 14 November 1807, and subsequently baptised on January 2nd, 1809 in Feltham, Middlesex, the daughter of Charles PLUNKET and Harriet (née VILLEBOIS). She is mentioned in her grandmother's will (Frances Villebois (née Read) as 'my Grand Daughter Harriet Plunket'. She married Charles GARDINER at Walcot, Bath on 22 May, 1826. The marriage was subsequently dissolved by Act of Parliament in 1837. She again married soon after on 22 May, 1837 to Thomas Pullen WARD in St Mary, Islington, Middlesex. The couple were living in North Street, Winkfield, Berks in 1851. She later died on 3 May, 1864 whist living at Elm Cottage, Golders Green (Feltham, Middlesex).

http://search.findmypast.co.uk/record?id=gbprs%2fb%2f907038895%2f1&highlights=%22%22. Will of Frances Villebois, TNA reference PROB 11/1630 f.171v line 31. http://search.findmypast.co.uk/record?id=gbprs%2fm%2f295062114%2f3&highlights=%22%22. 'An Act to dissolve the Marriage of Charles Gardiner Esquire with Harriet Maria Elizabeth his now Wife, and to enable him to marry again; and for other Purposes'. Private Act (Not Printed), 7 William IV & I Victoria I, c. 52 House of Lords archives PO/PB/1/1837/7W4&1V1n60, 1837; *The Sessional Papers of the House of Lords,* 2 March 1837. 1851 census (TNA reference HO107/1694 f.552 p.4). GRO Index to Deaths registered in Q2 1864, Registration District: Hendon, Volume: 3a ,Page: 77 on which she is recorded as 'Harriet Elizabeth Mary'.

15. Frances GARDINER

Frances Charlotte was born in Childerton, Amesbury, 6 December, 1828, the daughter of Charles GARDINER and Harriet (née PLUNKET). She was married at Walcot, Bath on 24 May 1849 to James Richard LYSAGHT. The couple lived at Mowbray Lodge , Kirkby Malzeard, Yorkshire and are recorded with their children in the censuses of 1851 and 1861. They emigrated to New Zealand in 1874. She died in Hawera, New Zealand on 15 September, 1907.

1851 census (TNA reference HO107/1684 f.105 p.8). 1861 census (TNA reference RG09/3198 f.4 p.2). British Library 19th century newspapers online, *The Standard,* 26 May 1849, 5 July 1850, 9 Feb 1852, 2 May 1855. *The Morning Post*, 19 Oct. 1863. Online biography in rootsweb by great-granddaughter. New Zealand Registration of Births, Marriages and Deaths, death registration number 1907005240 on which she is recorded as having died age 76 and as having lived in New Zealand 33 years.

16. Sophia LYSAGHT

Sophia Augusta was born at Mowbray Lodge, Kirby Malzeard, Yorkshire, on 18 December 1861, the daughter of James LYSAGHT and Frances (née GARDINER). Birth registered in 1st quarter 1862. She emigrated with her parents on the "Crusader" to Lyttelton, Canterbury, New Zealand in 1874. She married Francis Edward MOORE, a sheep farmer, son of Edward Moore, Rector of Frittenden, and Lady Harriet Janet Sarah Montagu-Scott in 1884 at Taranaki, New Zealand. Her mother-in-law was the daughter of Charles William Henry Montagu-Scott, 4th Duke of Buccleuch and 6th Duke of Queensberry and Harriett, daughter of the 1st Viscount Sydney of St Leonards. She died on 26th March 1945 in New Zealand.

Her birth was announced in *The Morning Post*, 21 Dec. 1861. GRO Index to Births registered in Q1 1862. District: Ripon, Vol. 9a,Page: 71, curiously she was register as name 'unknown' presumably because they had not yet decided upon a name. Online biography in rootsweb by granddaughter. New Zealand Registration of Births, Marriages and Deaths, marriage registration number 1884003384. This records her age at the time of marriage as 22 and confirms her birthplace as Kirby Malzeard, Yorkshire, and her parents being Frances Charlotte Gardiner and James Richard Lysaght. *Burke's Peerage*, 116th edition (1999), vol 1, p.410. New New Zealand Registration of Births, Marriages and Deaths, death registration number 1945/16922.

17. Marjorie MOORE

Marjorie Frances was born on 1st January, 1891 in the Chatham Islands, New Zealand, the daughter of Francis MOORE and Sophia (née LYSAGHT). She married George Frederick WHITEHORN, son of William Frederick Whitehorn on 15 January, 1916 in Hawera, New Zealand. Her marriage certificate gives her occupation as masseuse, and that of her husband as Bank Officer. The marriage was subsequently dissolved in 1935. She died in 1954 after having moved to England.

New Zealand Registration of Births, Marriages and Deaths, death registration number 1891/17123. New Zealand Registration of Births, Marriages and Deaths, marriage registration number 1916003328, this records her age at the time of the marriage as 25 and confirms her birthplace as Chatham Island, and her parents being Sophia Augusta Lysaght and Francis Edward Moore. Marriage dissolved in the Supreme Court, Wellington, 15th October, 1935. GRO Index to Deaths registered in Q3 1954, Age 63, Registration District: Kensington, Volume: 5c, Page 819.

18. Gabrielle WHITEHORN

Gabrielle Montagu was born in 1928 in New Zealand, the daughter of George WHITEHORN and Marjorie (née MOORE). She married Milton Edwin DULDIG in 1957 and lived in New Zealand, Australia and the UK. She died in 2004 at Guildford, Surrey. Wendy Elizabeth was her only daughter.

GRO Index to Deaths registered in Q4 2004, Registration District: West Surrey (761/1), 096/1204.

Supplementary Note 2h

Paternal line genealogical work for this project

Using *Burkes Peerage* as a starting point, since this is generally accepted as the 'authoritive' guide to the lineage of peers of the realm, Schürer traced, and was able to locate and contact, five distantly related individuals whose lineage could be traced back through an unbroken male line to Richard III – up through the Dukes of Beaufort, the Marquisses and Earls of Worcester, the Dukes of Somerset, to John of Gaunt and Edward III, and then back down through Edmund, Duke of York (John of Gaunt's brother) to Richard. These individuals have kindly taken part in the study.

It is important to note two events in this lineage, where sons were born illegitimate and later legitimized. The first of these is John Beaufort (1373-1410), 1st Earl of Somerset, the eldest of four illegitimate children of John of Gaunt and his mistress, Katherine Swynford. Katherine (née de Roet) married Hugh Ottes Swynford a knight in the service of John of Gaunt (thought to have died in 1372), and was governess to John of Gaunt's daughters before becoming his mistress. Katherine and John of Gaunt later married, Katherine becoming his third wife.

The second of these is Charles Somerset (c. 1460-1526), 1st Earl of Worcester, who was the illegitimate child of Henry Beaufort, 3rd Duke of Somerset and Joan Hill. Little is known about Joan Hill.

For a detailed genealogy please see Supplementary Figure 2. In relation to this figure, it is important to note that John Beaufort, 3rd Earl of Somerset, grandson of John of Gaunt, was created Duke of Somerset (1st Duke) in 1443. This technically expired with his death in 1444. His younger brother, Edmund, was later created Duke of Somerset in 1448. As this was a separate creation he was also technically the 1st Duke of this creation, however, many sources refer to him as the 2nd Duke. Subsequently his son Henry inherited the title under the same creation, thus technically he was the 2nd Duke under the second creation, yet is often referred to as the 3rd Duke.

Supplementary Note 3

The DNA analysis in relation to previous work

The DNA data would form just one strand of evidence for the identification of any putative remains of Richard III. This evidence would have to be taken alongside all other evidence: genealogical, archaeological, osteological and radiocarbon dating evidence.

In 2003, Belgian colleagues carrying out research into the remains of Margaret of York met John Ashdown-Hill at a conference and suggested tracing a living female-line relative of Margaret in order to provide a comparator for any mitochondrial analysis. John Ashdown-Hill, with the help of others, traced Joy Ibsen and her family, (see section above) whom could act as a comparator should any putative remains of Richard III be found. Joy Ibsen had two short sections of her mtDNA (HV1 and HV2) sequenced in 2006[29].

The sequencing of two small sections of Joy Ibsen's mtDNA control region was carried out a number of years ago and the results published in a non-scientific journal without peer review[29]. With the availability of current samples and recent technological advances allowing for much higher resolution typing, we started the genetic analysis for the current Richard III project from scratch, using DNA kindly provided by Michael Ibsen (the son of Joy Ibsen, who died in 2008. Michael Ibsen is Richard III's nephew, 16 times removed), and a second individual, Wendy Duldig (Wendy Duldig is Richard III's niece, 18 times removed; Wendy is Michael's 14th cousin, twice removed), whom Schürer and his team traced in 2012. It also included DNA analysis of five male line relatives identified by Schürer in 2012/13 using standard genealogical sources (see above).

Supplementary Note 4

Hair and eye colour

Over the last few years, the genetics behind physical appearance and its prediction for use in forensic and anthropological work has progressed immensely[30,31]. At present it is possible to predict categorical eye and hair pigmentation using tools such as IrisPlex[32] and HIrisPlex [33] for example, with correct predictions combining hair and eye colour in 3 out of 4 cases[34]. However, it is currently not possible to predict individualised continuous colour as even more genes and variants responsible for these traits have yet to be found that go beyond the 24 SNPs currently used in HIrisPlex. Figure 2a (Main text) represents a proposed selection of possible eye and hair images deduced from the genotype profile of Richard III. These images are taken from a database of individuals with similar final eye and hair colour prediction probabilities, hence give an indication of the possible range of pigmentation colours that Richard III may have had but not precise colours. The high probability of blue eye colour at 0.955 is an extremely high indicator that Richard III indeed had an overall blue eye colour; however the precise amount of differing pigment within the eye cannot be predicted (i.e. there is a chance of minor heterochromia yielding a ring of brown/orange-yellow or white colour), it is unlikely that the overall perception of blue colour would have changed. Images a-d in the Figure give a few examples of eye colour associated with a probability >0.95, from blue-grey, white-grey to a cleaner blue. The chance of error for a categorical blue eye colour prediction with such a high blue probability using a set of over 3800 individuals from several European countries is less than 2% [35]. Figure 2a also gives a few examples of hair colour that may be possible with a range near Richard III's hair colour probability of blond at 0.771, with brown at 0.143. As with eye colour, it is currently not possible to individually predict a continuous hair colour from DNA, only categories such as blond, brown, red and black are possible with good accuracy levels. Therefore in this Figure, e-h are based on individuals from a database with similarly high blond probability predictions and give an indication of the colour range but not precise colours that is possible from Richard III's 24 SNP HIrisPlex profile. Figure 2a - e and f are a representation of the blond hair colour from two individuals that fall within the range of the high blond probability observed in Richard III's profile. An interesting point to note here is that there is a phenomenon that is not yet completely understood in hair colour genetics, which involves an age-dependent hair colour change i.e. blond as a child that changes to brown or even black as the individual approaches adulthood, possibly due to hormonal changes. This occurrence does not appear in all blond children however: for example this age-dependent change occurred in only approx. 10% of cases in a 350 individual database (data not published). However it is possible that Richard III may have been blond as a child, and is predicted as blond using the HirisPlex model but displayed a darker brown-black hair colour in adulthood. Therefore g and h in the same figure represent individuals from a database with hair colour that is associated with an

age-dependent change i.e. blond as a child, that produce a high blond probability value using the HIrisPlex model but do not display blond as an adult, instead showing a brown-black hair colour. It is not possible to rule out this phenomenon and therefore it has been included in previous accuracy precision tests of HIrisPlex blond hair colour prediction[33,34]. Therefore Figure 2a e-h represents the full range of hair colours that may have been possible.

Supplementary Note 5

Portraits

There are no surviving contemporary portraits of Richard III. The earliest extant likenesses of the king post-date his death by at least twenty years (see F. Hepburn, Portraits of the Later Plantagenets, Woodbridge, 1986[36]). There are two portraits which vie for the title of earliest portrait, namely the Arched-Frame portrait, thought to date from the 1510s, and the Royal Collection portrait, thought to date from the same period.

The Arched-Frame portrait (please see main text for image kindly provided by the Society of Antiquaries of London) of Richard III, as well as being considered to be one of the earliest, is the one that is thought to have the least amount of overpainting. According to the conservation reports for the painting, “x-ray shows that a narrow band of white shirt originally visible at the base of the neck has been painted over. Otherwise the original surface, despite fairly heavy craquelure, is well-nigh intact."(Anooshka Rawden, Society of Antiquaries of London, personal communication). Dendrochronology dates the oak panel to 1510, with the same tree, probably from the eastern Baltic, providing a companion portrait of Richard's brother, Edward, and confirming it as one of the two earliest of all know portraits of King Richard III to have survived. Alterations to the painting were made at some point in its history, with slight adjustments around the mouth and to the length of the hair: conservation work of this minor over-painting reinstated the painting to its original appearance. This painting is very different from other paintings of Richard, which appear to derive from an original type represented by the portrait in the Royal Collection (many thanks to Anooshka Rawden, Society of Antiquaries of London, personal communication).

The Royal Collection Portrait: 1504-1520, Richard III

Image reproduced with kind permission of the Royal Collection Trust/ ©Her Majesty Queen Elizabeth II 2014.

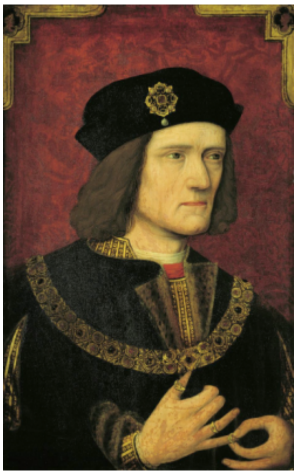

The portrait of Richard III in the Royal Collection is thought to date from between 1504 and 1520 on the basis of dendrochronological analysis. It was part of a set of early portraits of kings and queens recorded in Henry VIII's collection, commissioned either by him or his father, and probably painted following an earlier drawing or painting. At some point, the portrait was altered to show the king's proper right shoulder as being higher than the left one. It is also thought the artist may have altered the mouth and the colour of the eye (from brown to grey - for more information, see J. Scott 'The Royal Portrait: Image and Impact', London, 2010[37]). This depiction of Richard III seems to have served as the model

for most later portraits. For more information see:
http://www.royalcollection.org.uk/collection/403436/richard-iii-1452-85

Richard III (with broken sword) The Society of Antiquaries of London

Image reproduced with kind permission of the The Society of Antiquaries of London

The Broken Sword portrait was given to the Society of Antiquaries, London, in the same bequest as the Arched-Frame portrait described above. Dendrochronological analysis dates the oak panel upon which it is painted between 1523 and 1555. The proper left shoulder of the sitter is unnaturally enlarged and his hand is noticeably uncomfortable both in its pose and in its rendering. X-ray analysis of the painting has revealed under-drawing that shows a significantly deformed left arm. It appears as if adaptations to the king's costume and a reduction in the shoulder were both attempts to reduce and soften these features. It is very hard to determine when this may have occurred. Interestingly, this portrait differs strikingly from the Royal Collection portrait, but - like the Arched-Frame portrait - shows Richard with blue eyes and lighter hair. (many thanks to Anooshka Rawden, Society of Antiquaries of London, for information, personal communication)

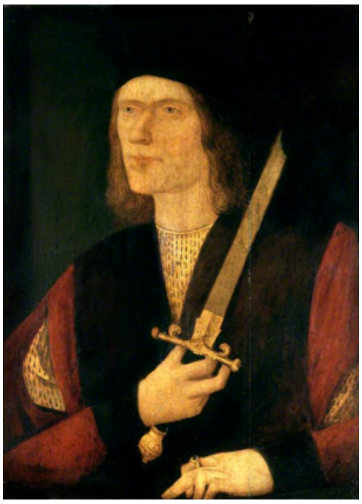

The portraits in Supplementary Figure 6 also all date from within ~120 years after Richard's death but appear to be copies of the portrait in the Royal Collection or its presumed prototype.

Supplementary Methods

Methods Summary

DNA was extracted from saliva samples from modern relatives of Richard III and all participants were recruited with informed consent following review by the University of Leicester Research Ethics Committee. All DNA work involving the modern relatives was carried out at the University of Leicester. Male-line relatives were typed using Promega PowerPlex®Y23 and for SNPs defining the main European Y haplogroups with a subset of the typing being confirmed at the Université Paul Sabatier. Female-line relatives were sequenced for the entire mitochondrial genome on an Ion Torrent PGM™ at the University of Leicester. DNA was extracted from teeth and bone from the ancient samples at the University of York and the Université Paul Sabatier, Toulouse. Library preparation and target enrichment were done at the University of York. Single-end 100-bp sequencing using a HiSeq 2000 (Illumina, CA, USA) was performed at the Copenhagen Sequencing Facility. Targeted sequencing of both modern and ancient DNA was also carried out at genomic technical platform PlaGe (Genopole, Toulouse, France) and at the Protein Nucleic Acid Chemistry Laboratory at the University of Leicester. Base calling was done using custom pipelines. Details of each step are to be found below.

Excavation and sample collection

Skeleton 1 was excavated over two days by Joanna Appleby (with Turi King on day 1) under clean conditions as per Yang and Watt, 2005[38]. Digging implements were cleaned in 10% bleach and those excavating wore Tyvek coverall suits (DuPont, UK), face masks and double gloves, the outer pair of which was changed regularly. The skull, mandible and right femur were loosely wrapped in clean aluminium foil, placed into clean individual sample bags and brought to the archaeology department to be stored in a lab where no human specimens had been kept or genetic work carried out previously. Under clean conditions, four teeth (right molar 2, left molars 1, 2 and 3) that could be used as potential DNA sources were removed from the mandible and placed into clean sample bags. The femur was left intact and no sample taken until in a dedicated ancient DNA lab. All samples were stored and subsequently sampled in a class 1000 vertical laminar flow clean room in the Space Research Centre under the care of John Holt. Using planetary protection controls to mitigate cross contamination risk, this facility is usually reserved for the assembly of space components and has never been used for biological research. Under aseptically controlled conditions, teeth were placed into bleach-washed and autoclaved sterilized glass containers (with PTFE seals) and the femur placed into a bag constructed from sterile Tyvek coverall in order to allow it to dry. All samples were stored without light at a constant 4°C until processed. Modern samples were collected using Oragene DNA Collection Kits (DNA Genotek). Everyone involved in the excavation at the Grey Friars site, the clean lab in Leicester and those involved in the labwork had their mitochondrial and, for males, Y chromosomes typed. DNA was extracted from saliva samples and all participants were recruited with informed consent.

DNA extraction - ancient sample

DNA was ultimately extracted from four teeth and one bone (femur) sample. All procedures were performed in dedicated ancient DNA laboratories at the University of York and the Université Paul Sabatier, Toulouse with appropriate contamination precautions in place. Two extraction blanks were included and treated exactly as if they were extracts throughout the whole process. PCRs and library experiments also included further blank controls. See Supplementary Table 1 for extractions.

Toulouse

The tooth (RM2) was cleaned with bleach, rinsed with deionized water and, finally, irradiated under UV light for 45min on each side. Tooth powder was obtained by grinding the tooth under liquid nitrogen with a 6870 SamplePrep Freezer Mill® (Fischer Bioblock, Illkirch, France). DNA was extracted from the tooth powder (see Supplementary Table 1) by incubating the powder at 50°C in a lysis buffer containing 500µL of EDTA (0.5µM), 50µL of proteinase K (20mg/mL) and 5µL of DTT (1M). This solution was then purified using the minElute PCR purification kit (Qiagen, France) in an elution volume of 40ul.

York

A short (~2x0.5cm) segment of bone was cut from the femur using a Dremel multitool and a disposable sanding disk previously sterilized. The teeth and bone were UV-irradiated (10 minutes each side) before their surfaces were cleaned using a Dremel multitool. For each sample, a new disposable disk on a Dremel multitool and subsequently UV- irradiated for 20 minutes on each side. Tooth powder was obtained by grinding the samples with mortar and pestle. DNA was extracted as per Rohland et al. 2009[39] and eluted in a final volume of 50ul. In the first instance, DNA was extracted from a portion of the tooth root (LM1) and part of the bone fragment only. When this proved insufficient to carry out the analysis required for whole mitochondrial genome sequencing, the other teeth were used to extract DNA.

Sex-Typing Assay - ancient sample

(Toulouse)

PCR primers for amplification of the SRY fragment were designed from previously published SRY sequences, and aligned using BioEdit v.7.0.5.3. PCR primers for co-amplification of UTX and UTY homologous regions were designed from conserved UTX/UTY regions of previously published UTX and UTY sequences, and aligned using the same software. The resulting conserved regions for each gene were used for primer design using Primer3 software v.0.4.0 to enable relatively small sized fragments of UTX and UTY to be co-amplified from samples likely to contain degraded DNA. Forward primers were 5'-labeled with a fluorescent FAM-dye (Invitrogen) to allow detection by capillary electrophoresis. Primer sequences and amplicon sizes are shown in Supplementary Table 2[40].

UTX, UTY and/or SRY regions were simultaneously amplified in a final volume of 15µl consisting of 1X Multiplex PCR Master Mix (Qiagen, ref:206152), 0.5X Q-solution, 0.1-0.25µM primers and 3µl template DNA. Primer concentration for each primer is given in Supplementary Table S2. The cycling conditions consisted of a first denaturation step at 95°C for 5 min followed 35 cycles of denaturation at 95°C for 30 sec, annealing at 62°C for 90 sec and extension at 72°C for 30 sec with a final extension step at 68°C for 30 min.

PCR products were visualized using capillary electrophoresis detection. Samples were prepared using 2 µl PCR products then mixed with 0.2µl of internal size standard GeneScan-600LIZ (AB) and 8.8µl Hi-Di Formamide (AB). After denaturation at 95°C for 5 min, samples were run on an ABI 3730 Genetic Analyzer (AB) using POP-7 (AB). Data were analyzed using GeneMapper software v. 4.0 (AB).

Mitochondrial control region analysis - ancient sample

PCR amplifications (Toulouse and York)

Analysis of the first, second and third hypervariable segments (HV1, HV2, HV3) of the mtDNA control region was done by amplifying and directly sequencing overlapping fragments ranging from 153–250bp in size (http://forensic.yonsei.ac.kr/protocol/mtDNA-midi-mini.pdf). PCR conditions and primers for HV1 used in Toulouse are published in Gabriel et al. 2001 (HV1a: F15989, and HV1b: F16190/R16410) and Ivanov et al. 1996 (HV1a: R16239). For HV1 (York), HV2 and HV3 (both labs), primers used were those in mini-primer set available from http://forensic.yonsei.ac.kr/protocol/mtDNA-midi-mini.pdf. PCR

amplifications were performed in 20ul reactions volumes containing 1x AmpliTaq Gold buffer, 4mM MgCl2, 0.5 µM dNTP (each), 0.05 mg/ml BSA, 2U AmpliTaq Gold (Applied Biosystems), 1.5µM of each primer and 5µl of DNA extract. The PCRs consisted of an initial denaturation of 9 minutes at 94°C, followed by 35 cycles of 94°C for 30sec, 52°C for 45 sec, and 72°C for 45 sec, followed by a final extension at 72°C for 5 minutes.

Cloning (Toulouse)

A selection of amplicons was used for cloning the PCR products. Post-PCR steps were performed in a post-PCR room dedicated to cloning and sequencing. PCR product bands visible on an agarose gel were extracted with MinElute Kit (Qiagen) and sequenced directly. The 15 µl of the remaining PCR products were purified and cloned into a pGEM®-T Easy Vector Systems (Promega). Vector DNA purifications for each clone were performed using QIAprep Spin Miniprep kit (Qiagen). For each PCR product, we have attempted to obtain at least ten clones. Cloned products were amplified using the SP6 and T7 primers to obtain both strands of the cloned PCR products (pGEM®-T Easy Vector, Promega).

Sequencing (Leicester and Toulouse)

Sequencing was carried out using Big-Dye Terminator V3.1 cycle sequencing kit (Applied Biosystems). The sequence reaction products were purified using QuickStep2 PCR Purification Cartridges (EdgeBio) in Leicester or Sephadex G-50 Fine (Sigma-Aldrich) in Toulouse. They were sequenced by capillary electrophoresis on an ABI Prism 3730 Genetic Analyser (Applied Biosystems) at the Protein Nucleic Acid Chemistry Laboratory at the University of Leicester or at the genomic technical platform PlaGe (Genopole, Toulouse, France).

Whole mitochondrial genome, Y-SNP and HIrisplex typing - ancient sample

Library preparation (York)

Libraries were built following Meyer and Kircher (2010)[41], with the exception that the first filtration step between the blunt end repair and the adapter ligation was substituted by heat inactivation of the enzymes (Fortes and Paijmans, 2014[42]; Bollogino et al., 2013[42]). Libraries were then amplified in two rounds, using AmpliTaq Gold DNA Polymerase. First, each library was amplified in eight parallel PCRs of 15 cycles, using the primers IS7 and IS8 [43]. The PCR products were pooled and purified using MinElute columns. Subsequently, the library was amplified in a second run of 8 PCRs of 10 cycles, using as template 2.5 ul of the eluted volume and the primers IS4 and P7-indexing.

Probes and Capture (York)

We designed two microarrays, one for the mtDNA-enrichment and another one for nuclear SNP enrichment. DNA enrichment was performed by hybridization capture using the Agilent 244k DNA SureSelect™ microarray (Agilent, Böblingen, Germany). For the first mitochondrial capture, we used the Cambridge reference sequence (CRS, NC_012920) as template for designing the probes, after excluding repetitive motives (Repeatmasker, www.repeatmasker) to capture mitochondrial sequences from

libraries generated from LM1 root and bone**.** When this provided insufficient coverage, a second capture was carried out. For this second capture, probes generated from the whole mitochondrial genome sequence of mitochondrial lineage 1 and 2 (ML1 and ML2) were used to capture mitochondrial sequences from libraries generated from LM1 (rest of tooth), LM2 and LM3. The mitochondrial sequence was cut down to 60 bases length, with 1-base pair tiling to obtain the probes. For the nuclear capture, Y chromosome probes were designed to cover the SNPs relevant to the major European lineages[44]. Further probes were designed to cover the SNPs relevant to the HIrisplex[33] markers. Rs numbers were used to pull out the SNP and surrounding sequences from NCBI. Probes were generated by placing the SNP in the middle of a 60bp probe and then using a sliding window to generate probes with the SNP placed at 5bp intervals ranging from ~10bp in on one side through to ~10bp in on the other side of the probe. See Supplementary Tables 5 and 9 for SNPs. These two sets of probes (mitochondrial and SNPs) were used separately to fill the two different microarray designs of a 1x244 k format.

For each microarray, the capture protocol was performed following Hodges et al. (2009)[45] with the modifications proposed by Zhang et al. (2013)[46]. After the first capture, the eluted volume from each of the arrays was amplified in 16 parallel PCRs of 20 cycles, using primers IS5 and IS6. Subsequently, the PCR products were pooled and purified using four MinElute columns (Qiagen) with a final elution volume of 20 µl each. The total 80 µl of amplified libraries diluted in EB were used as template for a second run of capture, which has been shown to increase the efficiency of the enrichment procedure[46,42]. The libraries were pooled in equimolar quantities and sequenced on two lanes of the Illumina HiSeq 2000 platform at the sequencing facility of the University of Copenhagen, Denmark.

Processing and mapping of raw sequence data

(Leicester and York)

Raw reads processing

The libraries were sequenced on a HiSeq2000 Illumina platform run in 100 SE mode. The raw reads from each library were sorted based on the 6 nucleotide index used during library preparation. Only reads with a 100% match to the index were selected for further analyses.When reads are shorter than the length of the sequencing (<100 bp), part of the P7 adapter sequence could be found at the end of the read. We used the software cutadapt v1.3[47] to trim the reads from any fragment of the P7 adapter. After trimming, reads shorter than 25 nucleotides were discarded from further analysis.

Mapping

The trimmed reads were mapped to autosomes and sex chromosomes from the human reference genome build 37 (GRCh37) and to the revised Cambridge Reference Sequence (rCRS, NC_012920.1) using bwa 0.7.5a-r405[48]. To improve mapping efficiency in ancient molecules, which are expected to be affected by deamination at the 5' and 3' positions, seed length was disabled (-l 1000)[49]. In each alignment, the output bam files were merged using SAMtools 0.1.19-44428cd[48] and PCR duplicates were removed subsequently. Furthermore, the mapped reads were filtered based on a mapping quality >29 and their alignment to unique positions along the reference sequence. Supplementary Table 7 summarizes the alignment statistics and mean depth of coverage of the mtDNA. See Supplementary Figure 3 for coverage of sample. We targeted a range of SNPs with known phenotypical/physiological effects. As this approach provided insufficient coverage we concentrated at verifying SNPs that provide phenotypic and Y chromosome information.

Variants calling and haplotype determination
Polymorphic positions were identified using SAMtools (samtools-0.1.19) and bcftools, specifying a haploid genome. Finally, vcfutils.pl was used to filter the list of variants according to a Phred-scaled genotype posterior probability quality > 20 and a read depth higher than 10. To avoid mis-calling because of the deamination pattern of ancient DNA molecules, all the polymorphic positions reported in the vcf output file were checked by eye. In the case of the mitochondrial genome, the assembly to the reference was visualized in Tablet[50], while the alignment of the reads containing the SNPs to the reference chromosomes was visualized using IGV[51]. Finally, the haplotype assignment (J1c2c3) was determined using Haplogrep (//haplogrep.uibk.ac.at)[52]. See Supplementary Table 6.

SNP typing by PCR

(Leicester, York and Toulouse)

The capture approach yielded insufficient coverage for all HIrisPlex and Y chromosome SNPs and therefore primers were designed to generate amplicons containing these SNPs as well as two SNPs which further define Y chromosome haplogroup G: M285 (G1) and P287 (G2)[44]. These were amplified as part of multiplex reactions following Römpler et al. 2006[53] or singleplex reactions (using 40 cycles and with no secondary amplification) and sequenced on the IonTorrent following library preparation using Ion PGM 200 Xpress Template Kit and PGM 200 Sequencing Kit. To increase coverage, singleplex PCR and sequencing of one marker (rs28777) was carried out according to Binladen et al. 2007[54]. See Supplementary Tables 5 and 9.

Typing of the haplogroup G defining SNPs (M201, M285 and P287) was repeated in Toulouse using singleplex PCRs following the protocol above. Sequencing of these PCRs products was carried out using Big-Dye Terminator V3.1 cycle sequencing kit (Applied Biosystems). The sequence reaction products were purified using Sephadex G-50 Fine (Sigma-Aldrich) and analysed by capillary electrophoresis on ABI Prism 3730 Genetic Analyser (Applied Biosystems) at the genomic technical platform PlaGe (Genopole, Toulouse, France).

Y-chromosomal haplotype analysis- ancient and modern samples

(Leicester, York and Toulouse)

Both for ancient and modern samples Y-chromosomal haplotypes were obtained using the PowerPlex® Y23 System (Promega) and analysed by capillary electrophoresis on ABI Prism 3730 Genetic Analyser (Applied Biosystems) at the genomic technical platform PlaGe (Genopole, Toulouse, France) and on a ABI Prism 3130xl Genetic Analyser (Applied Biosystems) at the University of Leicester. For skeleton 1, this was carried out on three separate extracts (RM2, LM1 and LM3) in two different ancient DNA labs (York and Toulouse). For the modern relatives this was carried out on two different extracts in two different modern labs (Leicester and Toulouse). See Supplementary Tables 3 and 4 and Supplementary Figure 3.

Y-chromosomal SNP analysis: modern samples

(Leicester and Toulouse)

Following determination of the Y haplotype for the modern male line samples, the predicted haplogroup was determined using Whit Athey's haplogroup predictor (http://www.hprg.com/hapest5/hapest5a/hapest5.htm?order=num). Binary markers covering these and related lineages were typed in two multiplexes by the SNaPshot minisequencing procedure (Applied Bio-systems) and an ABI3130xl Genetic Analyzer (Applied Biosystems) followed by confirmation using Sanger sequencing. Som1,2,4,5 were determined to be derived for R1b-U152. Som3 was determined to be Hg I (M170+, M223-, M253-)[44] derived, further confirmed by the lab in Toulouse. Following this, SOM1-5, were tested, for SNPs subdividing this clade[55] Z56, M126, Z36, Z192, M160 and L2 using Sanger sequencing in both labs. See Supplementary Figure 4.

Mitochondrial DNA analysis: modern samples

(Leicester)

Both samples were replicated twice.

Control region
Samples were taken using Oragene DNA Collection kits (DNA Genotek) and DNA extracted using two different methods: the Qiacube Blood and Body Fluid protocol (200ul with 200ul elution) and the Oragene protocol. These samples were sequenced twice in both the forward and reverse direction using two over-lapping primer sets (15973-296 and 16524-614) using BigDye Terminator V 3.1 (Applied Biosystems). No differences were found between replicates or between samples.

Whole mitochondrial genome - processing and analysis
Samples were then whole mitochondrial genome amplified following Meyer et al. 2007[12]. PCR amplicons were sequenced on an Ion Torrent PGM Sequencer on an Ion314 Chip. Libraries were prepared using the Ion Xpress™ Plus gDNA Fragment Library Preparation kit while the template preparation and the sequencing were carried out using the Ion PGM™ 200Xpress™ Template Kit and the Ion PGM™ 200 Sequencing Kit respectively. An output of 77.5 megabases (Mb) was obtained (of which 66.51 Mb are Q20) with an average read length of 169bp. Raw reads were mapped back to the revised Cambridge Reference Sequence (rCRS, NC_012920.1) using TMAP software included in the Ion Alignment plugin 3.2.1 (Torrent Suite™ Software 3.2.1) on the Ion Torrent server. Duplicate reads removal and variant calling were performed using SAMtools 0.1.19[48] and local realigning was carried out with The Genome Analysis Tool Kit (GATK)[56]. The average coverage for the two replicates for ML1 and ML2 is 258x and 264x respectively. Variant sites were filtered for Base Quality (BQ) 20, Mapping Quality (MQ) 50 and Depth of Coverage 30. One heterozygote was also called with low quality values. All the polymorphic positions reported in the vcf output file were manually checked using IGV 2.3[51]. The one heterozygote site (9546), found at the end of a poly-G tract, was characterised by low sequencing quality and was called as heterozygous in both modern samples. This was checked manually as well as being Sanger sequenced and found to be monomorphic. From a manual inspection, it appears to be the result of an alignment problem. After removal of the heterozygote, 33 polymorphic sites were retained. All these sites have been manually checked and confirmed by Sanger sequencing. See Supplementary Table 6.

While the mutation rate for position 8994 has not been estimated, examination of the data of Soares et al (2009)[57] places it in the top 8% of sites in terms of substitution rates. Furthermore, the 8994A is found in 431 of 19299 full mitochondrial genomes across 108 haplogroups, indicating that substitutions are highly recurrent at this position.

Medical relevance of Mitochondrial SNPs

Two SNPs were found to have possible medical relevance. Both of these two variants have been associated with Parkinson's disease, with one being associated with early onset and the other conferring resistance to the disease. See Supplementary Table 9.

Contamination control and quantification

Modern DNA contamination of the ancient remains was controlled for by the following methods:
1. Excavation was carried out under clean conditions (see above)
2. Samples were stored in clean labs and ancient DNA work carried out only in dedicated ancient DNA facilities.
3. Separate ancient samples were processed in separate labs in order to replicate results.
4. All lab members and excavation participants had their mtDNA typed and Y chromosome typing was carried out on all men involved. None had a matching mtDNA or Y chromosome type.

As evidence against significant contamination, DNA analysis shows a perfect mtDNA match to ML1 and a single base difference with ML2. It also shows a clear Y-STR haplotype which has been replicated using a number of extracts generated independently in two separate labs.

As a further test to determine whether there was evidence of any substantial contamination we investigated the substitution pattern in our mitochondrial reads. For this test, the mtDNA tree Build 16 (19 Feb 2014) at www.phylotree.org was queried and 251 SNPs which designate all main mitochondrial lineages, other than that of Richard III, were tabulated. These sites were then manually checked to look for reads which did not have the consensus sequence and were tabulated making note of the number of transitions from C>T or G>A, other transitions, transversions, insertion and deletions. Substitutions in reads can be due to sequencing errors, DNA damage (only C>T or G>A changes[58])or contamination. As contamination is unlikely to derive from African lineages, 251 SNPs from the main branches within lineage L0 were chosen and the exercise repeated to give a baseline for sequencing error and DNA damage. Controlling for differences in coverage, a two-tailed, independent t-test was carried out on the two datasets with no significant differences found between them ($p>0.05$).

Statistical Analysis

In the first such analysis of its kind, we bring together the genetic and genealogical evidence described above with previously-reported non-genetic evidence to come to an overall conclusion about the identity of Skeleton 1. Briefly, historical records (see above) tell us that, in life, Richard was described by a contemporary as having one shoulder higher than the other, that he was aged 32 at the time that he

was violently killed at the Battle of Bosworth, and that he was buried in the choir of the church of the Grey Friars in Leicester in 1485. In 2012, Skeleton 1, the remains of a male aged in his early 30s with scoliosis and perimortem battle injuries was discovered in the remains of the choir of the church of the Grey Friars site in Leicester with modelled radiocarbon dates bracketing 1485. Finally, we find a DNA match with modern maternal-line relatives.

To combine all the evidence, and taking a conservative approach at each step, we computed a likelihood for each item of observed evidence under each of two opposing hypotheses:

Hypothesis 1 (H1): Skeleton 1 is Richard III, and
Hypothesis 2 (H2): Skeleton 1 is not Richard III.

Logically, the likelihood for each item can depend on the items previously assessed, but for the data types discussed below it is reasonable to assume that all the different items of evidence are independent. Then, the joint likelihood of all the evidence is obtained by multiplication of the individual likelihoods under each hypothesis. The weight of evidence for H1, called the likelihood ratio (LR), is then given by the
ratio of the likelihood under H1 to that under H2. In the following we will say that an assumption is "conservative" if it reduces the LR.

The LR can be converted into a probability that H1 is true, given a prior probability. We take as starting point the moment that Skeleton 1 was first observed and recognised as a human skeleton, but before any assessments of age, sex, state of health and cause of death were made. At that point there was substantial evidence that a skeleton found in what is believed to have been the location of the Leicester Grey Friars choir could be that of Richard III. All of the information available at the time that Skeleton 1 was unearthed, including its precise location and the nature of the grave, is regarded for this analysis as background information that can inform the prior probability (and all likelihood calculations). Based on that information we believe that a sceptical observer could not reasonably have assigned a prior probability less than 1/40. This value was proposed in a previous analysis (http://rationalgareth.com/), based on what we judge to be sceptical assessments. On the other hand, the highest probability that could be justified by the prior evidence might be 1/2.

The likelihoods under H2 for many data types are based on count data, for example the number (x) of matching mtDNA sequences observed in a sample of n individuals. The ratio x/n is an unbiased estimator of the fraction θ of that mtDNA sequence in the population from which the sample was obtained, but it can have unsatisfactory properties if x is small. Generally the smaller is θ, the stronger the evidence against H2, so we avoid understating its value by using a uniform prior for θ. The posterior mean for θ is then $(x+1)/(n+2)$, which exceeds x/n whenever $x/n < 0.5$.

Below we give details of computation of the likelihoods for the various evidence types. We have used relevant, available data where possible. Inevitably subjective judgments are required for example about the relevant reference populations, and about the probabilities of error in reported facts. As far as seemed possible and reasonable, we have strived to be conservative in our approach.

Radiocarbon data:
An mean uncalibrated radiocarbon date of 437 ±13 was obtained from two samples of human rib bone

[59]. Calibration was performed using OxCal v4.2 (https://c14.arch.ox.ac.uk/) bounding between 1227±1.5 A.D. (representing the range of possible dates when the building of the Grey Friars friary was completed: 1224–30 A.D.) and 1538±.1 A.D. (when the dissolution of Grey Friars took place). Stable isotope analysis indicated a significant marine component to the diet and linear interpolation between $\delta^{13}C$ end members produced an estimate of 28% (±9). This was used to mix the Marine13 and IntCal13 calibration curves[60] during calibration, together with a local marine ΔR correction of -47±52 years, yielding an equal-tailed 95% confidence interval on the date of 1459.4 to 1536.3 A.D. For the likelihood of the radiocarbon date under H1, we took the fraction of the radiocarbon date distribution that fell into Richard III's lifetime (0.19), divided by the length of his lifetime (32.9 years), corresponding to an assumption that C14 isotopes were absorbed uniformly during his lifetime. Under H2, we used the same calculation applied to maximum lifespan of the Grey Friars friary (c1224-1538) (1 divided by 314 years). This yielded a likelihood ratio of 1.84, which represents limited support for H1.

Age and sex of skeleton:
Osteoarchaeological analysis of Skeleton 1 indicated that it was from a male aged between 30 and 34[59,61].We assumed the likelihood of this result under H1 to be 0.95 (to conservatively allow for the possibility of incorrect age and sex assignment). To evaluate the likelihood under H2 of finding a male skeleton aged between late 20s and early 30s, we considered data from the Carter Lane, St Mary Graces and Merton Priory sites in the WORD database (2014)[62] and at the site of the Leicester Grey Friars. Of the 706 skeletons with clear age and sex assignment, 127 were male in the age class 26 to 35. Using the posterior mean estimate for a population fraction described above yields a likelihood under H2 of (127+1)/ (706+2) = 0.18. The LR is then 5.25 (= 0.95/0.18), again representing limited evidence in favour of H1.

Presence of Scoliosis:
Skeleton 1 had severe idiopathic adolescent-onset scoliosis[63]. This might have lifted his right shoulder higher than his left, consistent with contemporary reports of Richard III's physical appearance (see above). However, two other known conditions, Erb's Palsy and Sprengel's deformity might also have given rise to this appearance. These are both very rare, with rate estimates for Erb's Palsy of 323 in 776,618 [64] and for Sprengel's (less than 1 in 200,000; see http://rarediseases.info.nih.gov/gard/7693/sprengel-deformity/resources/1). The Erb's Palsy rate is based on confirmed cases of congenital brachial palsy in Britain and Ireland (see Evans-Jones et al., 2003, Arch Dis Child Fetal Neonatal Ed;88:F185–F189), of which Erb's Palsy forms a subset, and so the rate is likely to be an over-estimate. For scoliosis, we used the observation of 5 cases among 1,476 skeletons, based on data from Roberts and Cox, Health & Disease in Britain (2000)[65].

Under H1, the above rates give an estimated probability of 0.90 of observing scoliosis given the description of Richard III's physical appearance (= the scoliosis rate divided by the sum of the three rates), which we multiplied by 0.95 to allow for the possibility that the recorded description was incorrect. Under H2, the likelihood of observing scoliosis was (5+1)/(1476+2) = 0.0041 leading to an LR of 212 (= 0.95x0.90/0.0041), providing moderately strong evidence in favour of H1.

Peri-mortem wounds:
Eleven peri-mortem wounds were identified on Skeleton 1, nine on the skull and two on the post-cranial skeleton. Two large wounds underneath the back of the skull will have been fatal [59]. All these wounds are consistent with written reports of Richard III being killed in battle. However, we assigned a likelihood

of 0.9 to these wounds under H1, to accommodate the possibility that the mode of Richard III's death had been exaggerated in historical accounts. To estimate the likelihood of peri-mortem wounds under H2, we took data on the rates of such wounds in the choirs of 8 priories dating to a similar period. We included only priory choirs, even though there is much additional data for other priory and church sites, as a choir is a prestige burial site and skeletons buried there may differ from those at other archaeological sites. The priory choirs considered (with observed proportions of skeletons with peri-mortem wounds) were: Exeter Dominican (0/4)[66], Gloucester Greyfriars (0/5)[67], Lewes Franciscan (0/6)[68], Ipswich Blackfriars (0/18)[69], Camarthen Franciscan (0/35)[70], St Mary Graces (1/17)(WORD database, 2014) [62 71], Merton Priory (0/6)[72] . This gave an overall likelihood under H2 of 2/93, giving an LR of 0.9x93/2 = 42, and so moderate support for H1.

Y chromosome:
The Y chromosome of Skeleton 1 did not match the presumed patrilinear relatives of Richard III. This could be explained by a false paternity event in one or more of the 19 putative father-son links between Richard III and Henry Somerset, 5th Duke of Beaufort. The Y chromosome results also indicate one further non-paternity event between Henry Somerset and his five living, presumed patrilinear descendants. False paternity rate estimates vary widely, as do the methods used to obtain them. To be conservative, we selected a published false paternity rate that was (1) low compared to other estimates, and (2) based on genealogical data. Larmuseau and colleagues (2014)[73] infer 8 false paternities in 936 putative father-son pairs, based on genealogical data. This may be an underestimate given since a number of Y chromosome STR mismatches are counted as due to mutation rather than false paternity[74], and is lower than any other published rate that we considered[75,76]. To this we add the non-paternity event in the 19 putative father-son links between Henry Somerset and five living male Somersets. Because this calculation is under H1 we don't use the posterior mean estimate, or the 19 putative father son links between Richard III and the Henry Somerset. Our false paternity rate is therefore (8+1)/(936+19). Over 19 generations this gives a probability of at least one false paternity event of $1-(1-9/955)^{19}$ = 0.16. Given that a non-paternity event must have occurred under H1, the population frequency of Skeleton 1's Y haplotype is the same under H1 and H2 and cancels out in the LR calculation. Thus the LR is 0.16, representing limited evidence against H1.

mtDNA:
The mtDNA sequences of Skeleton 1 and the presumed 19-meioses matrilinear relative of Richard III, Michael Ibsen, matched completely. A 21-meioses relative, Wendy Duldig, also matched except at one base (8994). However, the observation of a mutation is equally likely under H1 and H2 given the observed sequence of Michael Ibsen, and so cancels out in the LR calculation. Therefore, we only require likelihoods for the observation of the sequence shared by Michael Ibsen and Skeleton 1.

To obtain the likelihood under H1, we require an estimate of the mtDNA mutation rate, and in this case high estimates are conservative. As with the Y chromosome false paternity rate, estimates of mtDNA mutation rates vary widely, as do the methods used to obtain them. Parsons and colleagues (1997)[77] report 10 control region mutations in 327 generations using genealogical data. This is considerably higher than reported evolutionary rate estimates from ancient skeletons[78] possibly because of weak purifying selection. Because it is higher than other published estimates, and based on genealogical data, we used the Parsons et al (1997) data. The probability of no mutation in 19 meioses is then $(1-11/329)^{19}$ = 0.52.

For the likelihood under H2, we require the population fraction of Skeleton 1's haplotype. Although we obtained the complete mtDNA genome sequence from Skeleton 1, we identified little whole-genome comparison data from England. For this reason only the regions between positions 16093-16320 and between 00073-00188, for which there is suitable comparison data (albeit at low resolution) from England, were considered. This is a database which is an update of Röhl et al (2001)[79] supplemented with mtDNA sequences supplied by Roots for Real (Genetic Ancestor Ltd., Clare, Suffolk, UK). Restricting the observed data to these short sections of the control region under H2 is conservative, since the population fraction of the observed control region sequence cannot be less than that of the full mtDNA genome. The relevant reference population is over 500 years in the past, but due to the large population size over this period considered we expect population frequencies to have changed little over this time. The observed frequency of the haplotype is 0 among 1823 observed English mtDNA sequences covering the two regions above, to which we add one instance observed in Michael Ibsen. This approach is, again, conservative as he was sampled due to his known genealogical relationship to Richard III. Adding to this the observed sequence and using the posterior mean estimate gives 2/1826 = 0.0011, leading to an LR of 478 (=0.52/0.0011), representing moderately strong evidence for H1.

It is also worth noting that we chose to be further conservative and did not include the observation of no matches found in 26,127 European mitochondrial control region haplotypes (LR=6847) (http://empop.org)[80]. Female mobility among the European nobility is likely to have been much higher than for the general population, because of marriage practices relating to political alliance formation. Such practices would provide some justification for using the European mtDNA database, and so for considering the haplotype found in Skeleton 1 and Michael Ibsen to be extremely rare.

The data considered in each row of Supplementary Table 10 is indicated in the first column. The sceptical prior probability on H1 is 1/40, while the 50/50 prior probability is 0.5. The first row (using all evidence) is the one we propose should be relied on, the other rows are for illustration of the relative strengths of different components of the evidence. Excluding the mtDNA evidence is appropriate for the alternative hypothesis H2' that Skeleton 1 is a female-lineage relative of Richard III sufficiently close for no mtDNA mutation to have occurred. Excluding the Y evidence might be appropriate if it were considered that the documented patrilinear relationships were unreliable and so a non-paternity event was expected.

The LRs for different combinations of the evidence, and two posterior probabilities, are shown in Supplementary Table S10. Using all the evidence, the support for H1 is extremely strong with an LR of 6.7 million, so that our sceptic would be driven to the conclusion that the probability that Skeleton 1 is not Richard III is less than 1 in 100,000, while for those taking a 50/50 starting position that probability is much less than 1 in a million. Taking into account the conservative assumptions underlying our calculation described above, we regard this as establishing the truth of H1 beyond reasonable doubt.

Interestingly, although the "DNA match" reported by the University of Leicester on February 4, 2013 was popularly perceived to have "proved" that Skeleton 1 is Richard III, we find the genetic evidence supports H1 only moderately because the Y DNA evidence points in the other direction. Using our highly conservative approach, with a LR of 79 for mtDNA and Y evidence combined, our sceptic would not be convinced of H1 based on the genetic evidence alone. The non-genetic evidence weight of evidence is over a thousand times stronger than the genetic evidence, under the conservative assumptions outlined above. We note that if we are to remove the Y chromosome evidence, because of its susceptibility to

non-paternity events, the contribution of the genetic data strengthens considerably (LR= 478). Excluding the Y evidence might be appropriate due to the known possibility of non-paternity, especially given the legal and moral consequences of non-paternity cases in the past, should they have occurred, mean that they were likely to have been concealed.

For demonstration purposes, if we were to make use of the European mitochondrial DNA database (http://empop.org)[80] in our calculations then the mtDNA data alone gives strong support for H1 (LR=6847). Using all the evidence, the support for H1 is stronger still with our sceptic concluding that the probability that Skeleton is not Richard III is less than 1 in a million and for those taking a 50/50 starting position, is less than 1 in 100 million.

Although the possibility of Skeleton 1 being a close maternal relative has been excluded to the extent possible, as described above, there are likely to have been a large number of remote maternal-lineage relatives of Richard III, within about 50 meioses of him and hence plausibly sharing his full mtDNA sequence. These will be represented in the population from which the frequency data is obtained, and so there is no need to explicitly consider these remote, maternal-lineage relatives of Richard III as candidates to be Skeleton 1 (call this alternative hypothesis H2'). However, if a sceptic felt that H2' deserves special consideration as the primary alternative to H1, then the mtDNA evidence can be discarded, while all other evidence remains unaffected, resulting in an LR of 14,000, representing very strong evidence for H1 rather than H2'. We emphasise that this LR is for illustration only: since the mtDNA sequence of Skeleton 1 is absent from our population data, there is no particular evidence for Skeleton 1 to be a remote, maternal-lineage relative of Richard III, and, as far as possible, all known maternal relatives have been excluded (see above), we believe that it is reasonable to ignore H2'.

Supplementary References

1 Radzikowski, P. *Reisebeschreibung Niclas von Popplau, Ritter, Burtig von Breslau*. (1999).

2 Rous, J. *Joannis Rossi antiquiarii Wawicensis Historia Regum Angliae*. (Theatro Sheldoniano, impensis J. Flether and J. Pote, 1745 [1486]).

3 Vergil, P. *Historia Anglica*. (Scolar, 1972 [1555]).

4 Broadberry, S. N., Campbell, B. M. S. & van Leeuwen, B. *English medieval population: reconciling time series and cross sectional evidence* (2011).

5 Wrigley, E. & Schofield, R. *The Population History of England 1541-1871*. (Edward Arnold, 1981).

6 Bennett, M. *Hutchinson Dictionary of Ancient and Medieval Warfare.* (Helicon, Abingdon, 2005).

7 Hokes, E. M. & Wheeler, G. A Spanish account of the Battle of Bosworth. *The Ricardian* 2, 2 (1972).

8 Bennett, M. *The Battle of Bosworth*. (Sutton, 1985).

9 Winter, J. M. in *The upheaval of war: family, work and welfare in Europe, 1914-1918* (eds R. Wall & J. Winter) (1988).

10 Richardson, D. *Royal Ancestry*. (Genealogical Publishing Company, 2013).

11 Weir, A. *Britain's Royal Families: the complete genealogy*. (Vintage Books, 2008).

12 Stuart, R. W. *The complete known lineage of John of Gaunt, son of Edward III, King of England, and Queen Philiippa*. 4th edn, (Genealogical Publishing Company, 2002).

13 Gibbs, V. *The complete peerage of England, Scotland, Ireland, Great Britain and the United Kingdom extant or dormant / by G.E.C. [i.e. George Edward Cokayne] (St. Catherine Press, London, 1910-1936)*.
14 Mosley, C. *Burke's Peerage, Baronetage and Knightage*. (Burke's Peerage, Wilmington, Del, 2003).
15 Burke, J. & Burke, J. B. *A genealogical and heraldic history of the extinct and dormant baronetcies of England*. 2 edn, (John Russell Smith, 1964).
16 MAHD Heddle de la Caillemotte de Massue de Ruvigny Marquis de Ruvigny and Raineval, M. *The Plantagenet Roll of the Blood Royal, being a complete table of all the descendants now living of Edward III, King of England. The Anne of Exeter volume, containing the descendants of Anne-Plantegenet-Duchess of Exeter.* . Vol. 2 (T.C.& E.C. Jack, 1907).
17 Wagner, A. *The records and collections of the College of Arms*. (1952).
18 Norcliffe, C. B. *The visitation of Yorkshire in the years 1563 and 1564 made by William Flower, Norray King of Arms.* (Harlein Society, London, 1881).
19 Foster, J. *The Visitation of Yorkshire, made in the years 1584/5, by Robert Glover ... to which is added the subsequent Visitation made in 1612, by Richard St. George ... With several additional pedigrees, including "The Arms taken out of Churches and Houses at Yorkshire Visitation, 1584/5 ... "Sir William Fayrfax' Booke of Arms," and other heraldic lists, with copious indices.* (Private, 1875).
20 Clay, J. W. *Dugdale's Visitation of Yorkshire, [in 1665-66.] with additions.* (W. Pollard & Co., Exeter, 1899-1917).
21 Foster, J. *Pedigrees of the county families of Yorkshire*. (W. Wilfred Head, 1874-5).
22 Howard, J. J. & Crisp, F. A. *Visitation of England and Wales.* (Private, London, 1893-1921).
23 Townend, P. *Burke's genealogical and heraldic history of the landed gentry*. 18 edn, (Burke's Peerage, 1965-1972).
24 Perry, J. Katherine Roet's Swynfords: a re-examination of interfamily relationships and descent – Part 1. *Foundations: newsletter of the Foundation for Medieval Genealogy* 1, 122-131 (2003).
25 Perry, J. Katherine Roet's Swynfords: a re-examination of interfamily relationships and descent – Part 2. *Foundations: newsletter of the Foundation for Medieval Genealogy* 1, 164-174 (2004).
26 Kelly, H. A. Shades of Incest and Cuckoldry: Pandarus and John of Gaunt'. *Studies in the Age of Chaucer* 13, 121-140 (1991).
27 Hammond, P. in *Richard III: Crown and People* (ed J. Petre) (Richard III Society, 1985).
28 MAHD Heddle de la Caillemotte de Massue de Ruvigny Marquis de Ruvigny and Raineval, M. *Some Royal Descents. [Supplementary genealogical tables to the author's "Plantagenet Roll of the Blood Royal."]* (Private, 1909).
29 Ashdown-Hill, J. Alive and well in Canada: the mitochondrial dna of Richard III. *The Ricardian* 16, 113-132 (2006).
30 Kayser, M. & de Knijff, P. Improving human forensics through advances in genetics, genomics and molecular biology. *Nature Reviews Genetics* 12, 179-182 (2011).
31 Kayser, M. & Schneider, P. DNA-based prediction of human externally visible characteristics in forensics: motivations, scientific challenges, and ethical considerations. *Forens. Sci. Int. Genet.* 3, 154-161 (2009).
32 Walsh, S. *et al.* IrisPlex: a sensitive DNA tool for accurate prediction of blue and brown eye colour in the absence of ancestry information. *Forens. Sci. Int. Genet.* 5, 170-180 (2010).
33 Walsh, S. *et al.* The HIrisPlex system for simulataneous prediction of hair and eye colour from DNA. *Forens. Sci. Int.: Genetics* 7, 98-115 (2013).

34 Walsh, S. *et al.* Developmental validation of the HIrisPlex system: DNA-based eye and hair colour prediction for forensic and anthropological usage. *Forens. Sci. Int. Genet.*, 150-161 (2014).
35 Walsh, S. *et al.* DNA-based eye colour prediction across Europe with the IrisPlex system. *Forens. Sci. Int. Genet.* 6, 330-340 (2012).
36 Hepburn, F. *Portaits of the Later Plantagenets*. (The Boydell Press, 1986).
37 Scott, J. *The Royal Portrait: Image and Impact*. (Royal Collection Enterprises Ltd., 2010).
38 Yang, D. & Watt, K. Contamination controls when preparing archaeological remains for ancient DNA analysis. *Journal of Archaeological Science* 32, 331-336 (2005).
39 Rohland, N., Siedel, H. & Hofreiter, M. A rapid column-based ancient DNA extraction method for increased sample throughput. *Molecular Ecology Resources* doi: 10.1111/j.1755-0998.2009.02824.x (2009).
40 Cadamuro, V. C. *et al.* Determined about sex: sex-testing in 45 primate species using a 2Y/1X sex-typing assay. *Forens. Sci. Int. Genet.*, doi:10.1016/j.fsigen.2014.09.010 (2014).
41 Meyer, M. & Kircher, M. Illumina sequencing library preparation for highly multiplexed target capture and sequencing. *Cold Spring Harbour Protocols* 6 (2010).
42 Fortes, G. G. & Paijmans, J. L. A. in *Whole Genome Amplicfication* (ed T. Kroneis) (Humana Press, 2014).
43 Meyer, M. & Kircher, M. Illumina Sequencing Library Preparation for Highly Multiplexed Target Capture and Sequencing. *Cold Spring Harbour Protocols* 2010, doi:10.1101/pdb.prot5448 (2010).
44 Karafet, T. *et al.* New binary polymorphisms reshape and increase resolution of the human Y chromosomal haplogroup tree. *Genome Research* doi:10.1101/gr.7172008 (2008).
45 Hodges, E. *et al.* Hybrid selection of discrete genomic intervals on custom-designed microarrays for massively parallel sequencing. *Nature Protocols* 4, 960-974 (2009).
46 Zhang, H. *et al.* Morphological and genetic evidence for early Holocene cattle management in northeastern China. *Nature Communications* 4, doi:10.1038/ncomms3755 (2013).
47 Martin, M. Cutadapt removes adapter sequences from high-throughput sequencing reads. *EMBnetjournal, North America* 9 (2011).
48 Li, H. & Durbin, R. Fast and accurate short read alignment with Burrows-Wheeler transform. . *Bioinformatics* 25, 1754-1760 (2009).
49 Schubert, M. *et al.* Improving ancient DNA read mapping against modern reference genomes. *BMC Genomics* 13, 10.1186/1471-2164-1113-1178 (2012).
50 Milne, I. *et al.* Using Tablet for visual exploration of second-generation sequencing data. *Briefings in Bioinformatics* 14, 193-202 (2013).
51 Thorvaldsdottir, H., Robinson, J. & Mesirov, J. Integrative Genomics Viewer (IGV): high-perfomance genomics data visualization and exploration. *Briefings in Bioinformatics* 14, 178-192 (2013).
52 Kloss-Brandstatter, A. *et al.* HaploGrep: A fast and reliable algorithm for automatic classification of mitochondrial DNA haplogroups. . *Human Mutation* 32, 25-32 (2011).
53 Rompler, H. *et al.* Multiplex amplification of ancient DNA. *Nature Protocols* 1, 720-728 (2006).
54 Binladen, J., Gilbert, M. T., Campos, P. F. & Willerslev, E. 5'-tailed sequencing primers improve sequencing quality of PCR products. *Biotechniques* 42, 174-176 (2007).
55 Rocca, R. *et al.* Discovery of Western European R1b1a2 Y Chromosome Variants in 1000 Genomes Project Data: An Online Community Approach. *Plos One* 7, e41634 (2012).
56 McKenna, A. *et al.* The Genome Analysis Toolkit: A MapReduce framework for analyzing next-generation DNA sequencing data. *Genome Research* 20, 1297-1303 (2010).

57 Soares, P. *et al.* Correcting for Purifying Selection: An Improved Human Mitochondrial Molecular Clock. *American Journal of Human Genetics* 84, 740-759 (2009).

58 Hofreiter, M., Jaenicke, V., Serre, D., Von Haeseler, A. & Paabo, S. DNA sequences from multiple amplifications reveal artifacts induced by cytosine deamination in ancient DNA. *Nucleic Acids Research* 29, 4793-4799 (2001).

59 Buckley, R. *et al.* 'The king in the car park': new light on the death and burial of Richard III in the Grey Friars church, Leicester, in 1485. *Antiquity* 87, 519-538 (2013).

60 Reimer, P. *et al.* INTCAL13 and MARINE13 radiocarbon age calibration curves, 0-50,000 years cal BP. *Radiocarbon* 55, 1869-1887 (2013).

61 Appleby, J. *et al.* Perimortem trauma in King Richard III: a skeletal analysis. *The Lancet*, doi:10.1016/S0140-6736(14)60804-7 (2014).

62 WORD Database, Museum of London (ed Museum of London) (2014).

63 Appleby, J. *et al.* The scoliosis of Richard III, last Plantagenet King of England: diagnosis and clinical significance. *The Lancet* 383, 1944 (2014).

64 Evans-Jones, G. *et al.* Congenital Brachial Palsy: incidence, causes and outcome in the United Kingdom and Republic of Ireland. *Archives of Disease in Childhood Fetal & Neonatal Edition* 68, 185-189 (2003).

65 Roberts, C. & Cox, M. *Health & Disease in Britain from Prehistory to the Present Day*. (Sutton Publishing Ltd., 2000).

66 Loe, L. Princesshay, Exeter. A Note...remains. Unpublished summary report compiled for Exeter Archaeology/Exeter City Council (Oxford Archaeology South, 2011).

67 Ferris, I. Excavations at Greyfriars, Gloucester, in 1967 and 1974-5. *Transactions of the Bristol and Gloucestershire Archaeological Society* 119, 95-146 (2001).

68 Gardiner, M., Russell, M. & Gregory, D. Excavations at Lewes Friary 1985-6 and 1988-9. *Sussex Archaeological Collections* 134, 71-123 (1996).

69 Mays, S. A. The Mediaeval Burials from the Blackfriars Firary, School Street, Ipswich, Suffolk (Excavated 1983-1985) Report No. Report Number: 16/1991, (English Heritage, 1991).

70 Wilkinson, J. Excavations at Carmarthen Greyfriars 1983-1997, Analysis of Skeletal Remains, Vol. 1. (National Monuments Record of Wales, 2001).

71 Grainger, I. & Phillpotts, C. The Cistercian abbey of St Mary Graces, East Smithfield, London. (Museum of London Archaeology, London, 2011).

72 Miller, P. & Saxby, D. The Augustinain Priory of St Mary Merton, Surrey, Excavations 1976-90. (Museum of London Archaeological Service/English Heritage, London).

73 Larmuseau, M. H. D. *et al.* Low historical rates of cuckoldry in a Western European human population traced by Y-chromosome and genealogical data. *Proceedings of the Royal Society B* 280, 20132400 (2013).

74 Walsh, B. Estimating the Time to the Most Recent Common Ancestor for the Y chromosome or Mitochondrial DNA for a Pair of Individuals. *Genetics* 158, 897-912 (2001).

75 Foster, E. *et al.* The Thomas Jefferson paternity case. *Nature* 397, 32 (1999).

76 Jobling, M. A., Heyer, E., Dieltjes, P. & de Knijff, P. Y-chromosome-specific microsatellite mutation rates re-examined using a minisatellite, MSY1. *Hum. Mol. Genet.* 8, 2117-2120 (1999).

77 Parsons, T. J. *et al.* A high observed substitution rate in the human mitochondrial DNA control region. *Nature Genet.* 15, 363-367 (1997).

78 Rieux, A. *et al.* Improved calibration of the human mitochondrial clock using ancient genomes. *Molecular Biology and Evolution*, doi:10.1093/molbev/msu222 (2014).

79 Röhl, A., Brinkmann, B., Forster, L. & Forster, P. An annotated mtDNA database. *International Journal of Legal Medicine* Aug 115, 29-39 (2001).
80 Parson, W. & Dür, A. EMPOP-a forensic mtDNA database. *Forens. Sci. Int. Genet.* 1, 88-92 (2007).
